# Basigin deficiency prevents anaplerosis and ameliorates insulin resistance and hepatosteatosis

**DOI:** 10.1172/jci.insight.142464

**Published:** 2021-10-22

**Authors:** Akihiro Ryuge, Tomoki Kosugi, Kayaho Maeda, Ryoichi Banno, Yang Gou, Kei Zaitsu, Takanori Ito, Yuka Sato, Akiyoshi Hirayama, Shoma Tsubota, Takashi Honda, Kazuki Nakajima, Tomoya Ozaki, Kunio Kondoh, Kazuo Takahashi, Noritoshi Kato, Takuji Ishimoto, Tomoyoshi Soga, Takahiko Nakagawa, Teruhiko Koike, Hiroshi Arima, Yukio Yuzawa, Yasuhiko Minokoshi, Shoichi Maruyama, Kenji Kadomatsu

**Affiliations:** 1 Departments of Nephrology and; 2 Molecular Biochemistry, Nagoya University Graduate School of Medicine, Nagoya, Japan.; 3 Research Center of Health, Physical Fitness and Sports, Nagoya University, Nagoya, Japan.; 4 Departments of Legal Medicine and Bioethics and; 5 Gastroenterology and Hepatology, Nagoya University Graduate School of Medicine, Nagoya, Japan.; 6 Institute for Advanced Biosciences, Keio University, Tsuruoka, Japan.; 7 Center for Joint Research Facilities Support, Research Promotion and Support Headquarters, Fujita Health University School of Medicine, Toyoake, Japan.; 8 Division of Endocrinology and Metabolism, National Institute for Physiological Sciences, Okazaki, Japan.; 9 Department of Biomedical Molecular Sciences, Fujita Health University School of Medicine, Toyoake, Japan.; 10 Department of Nephrology, Rakuwakai Otowa Hospital, Kyoto, Japan.; 11 Department of Endocrinology and Diabetes, Nagoya University Graduate School of Medicine, Nagoya, Japan.; 12 Nephrology, Fujita Health University School of Medicine, Toyoake, Japan.

**Keywords:** Hepatology, Metabolism, Diabetes, Gluconeogenesis

## Abstract

Monocarboxylates, such as lactate and pyruvate, are precursors for biosynthetic pathways, including those for glucose, lipids, and amino acids via the tricarboxylic acid (TCA) cycle and adjacent metabolic networks. The transportation of monocarboxylates across the cellular membrane is performed primarily by monocarboxylate transporters (MCTs), the membrane localization and stabilization of which are facilitated by the transmembrane protein basigin (BSG). Here, we demonstrate that the MCT/BSG axis sits at a crucial intersection of cellular metabolism. Abolishment of MCT1 in the plasma membrane was achieved by *Bsg* depletion, which led to gluconeogenesis impairment via preventing the influx of lactate and pyruvate into the cell, consequently suppressing the TCA cycle. This net anaplerosis suppression was compensated in part by the increased utilization of glycogenic amino acids (e.g., alanine and glutamine) into the TCA cycle and by activated ketogenesis through fatty acid β-oxidation. Complementary to these observations, hyperglycemia and hepatic steatosis induced by a high-fat diet were ameliorated in *Bsg*-deficient mice. Furthermore, *Bsg* deficiency significantly improved insulin resistance induced by a high-fat diet. Taken together, the plasma membrane–selective modulation of lactate and pyruvate transport through BSG inhibition could potentiate metabolic flexibility to treat metabolic diseases.

## Graphical abstract

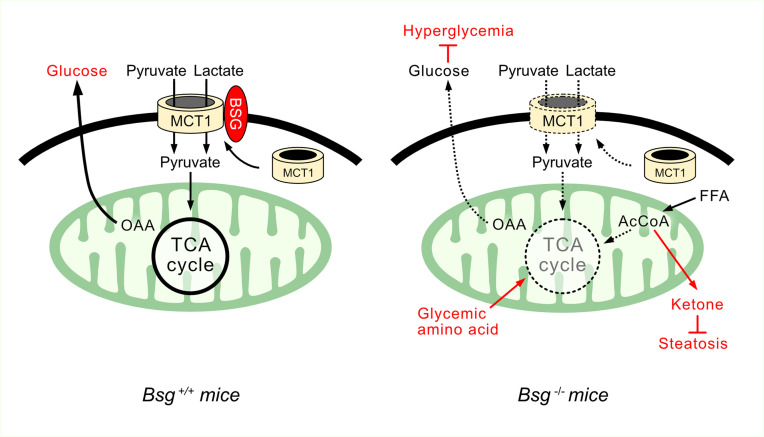

## Introduction

Gluconeogenesis from lactate, pyruvate, and amino acids is a critical physiological process for the maintenance of energy homeostasis during periods of nutrient deprivation (1, 2). In particular, the liver plays a crucial role in regulating gluconeogenesis while also performing ketogenesis and lipogenesis (3). Despite being essential for survival, gluconeogenesis — when activated in excess — can result in persistent hyperglycemia with devastating consequences, including chronic kidney disease and cardiovascular events. The pathogenic mechanism of excessive gluconeogenesis involves an impaired balance between the influx and efflux of tricarboxylic acid (TCA) cycle intermediates (1, 4–6). In contrast, insulin suppresses gluconeogenesis and promotes de novo lipid synthesis. In severe metabolic disorders associated with insulin resistance, insulin fails to suppress gluconeogenesis in the liver while the induction of hepatic lipogenesis is sustained, eventually leading to both hyperglycemia and hyperlipidemia (5–9). Thus, metabolic syndrome may be substrate driven while also facilitated by alterations in insulin signaling. Indeed, the depletion of TCA cycle intermediates and decreases in gluconeogenesis have been shown to ameliorate insulin resistance (6).

Both lactate and pyruvate promote endogenous glucose production as the precursor of hepatic and renal gluconeogenesis; the elevation of circulating lactate levels, therefore, is a relevant predictor of type 2 diabetes incidence in epidemiological studies (10–12). Proton-linked monocarboxylate transporters (MCTs) 1–4 facilitate the movement of monocarboxylates, such as lactate, pyruvate, and ketone bodies, across the plasma membrane, each having a different tissue distribution. MCT1 is expressed ubiquitously and is required for the influx of these substrates depending on their metabolic state, whereas MCT4 is prominent in various cells with a high glycolytic rate, such as white muscle, suggesting it is expressed where lactic acid efflux predominates (13, 14). MCT3 expression is more restricted to retinal pigment epithelium (15). The activity of these MCTs requires an accessory glycoprotein, basigin (BSG), that enhances their functional cell membrane expression by facilitating processes such as proper folding, stabilizing, and trafficking (16, 17). Notably, MCTs also contribute to the import and export of monocarboxylates across the mitochondrial membrane. In contrast, MCT2, which is capable of maintaining substrate uptake at low substrate concentrations in testis and renal proximal tubules, requires embigin and neuroplastin, but not BSG (18). The cascade involving MCTs and BSG might be a critical determinant for the maintenance of energy homeostasis, which is essential for viability. Improved understanding of the cellular metabolism of lactate and pyruvate via the MCT/BSG complex would be of great value, as these processes have large impacts on surrounding metabolic networks, including ketogenesis and lipogenesis, in the liver, kidneys, and skeletal muscles.

BSG, a highly glycosylated transmembrane protein belonging to the immunoglobulin superfamily, is widely expressed in hematopoietic cells, endothelial cells, and epithelial cells in a variety of organs, particularly in the liver and kidneys (16). A wide range of BSG binding partners have been described to date, including caveolin, cyclophilin, MCTs, and BSG itself (19, 20). Given the ubiquitous effects of lactate and pyruvate in cellular bioenergetics, identification of the physiologic role of the MCT/BSG complex has enormous potential for the treatment of human disease. To date, several studies using immortalized cancer cell lines have demonstrated that BSG may contribute to tumor invasion and metastasis by inducing further metabolic reprogramming, in addition to contributing to tissue remodeling via activation of matrix metalloproteinases (17, 19, 21). However, cancer cells typically express BSG to high levels, making such lines less than ideal for studying the physiological role of BSG in energy-related metabolic circuits. Therefore, the current study employed *Bsg*-deficient (*Bsg^–/–^*) mice and primary cultured hepatocytes. We demonstrate the abolishment of MCT1 from the plasma membrane in *Bsg*-depleted cells and highlight the position of the MCT/BSG complex at the crucial intersection of cellular metabolism in both physiological and pathological settings.

## Results

### Intracellular localization and function of BSG.

**Figure 1:**
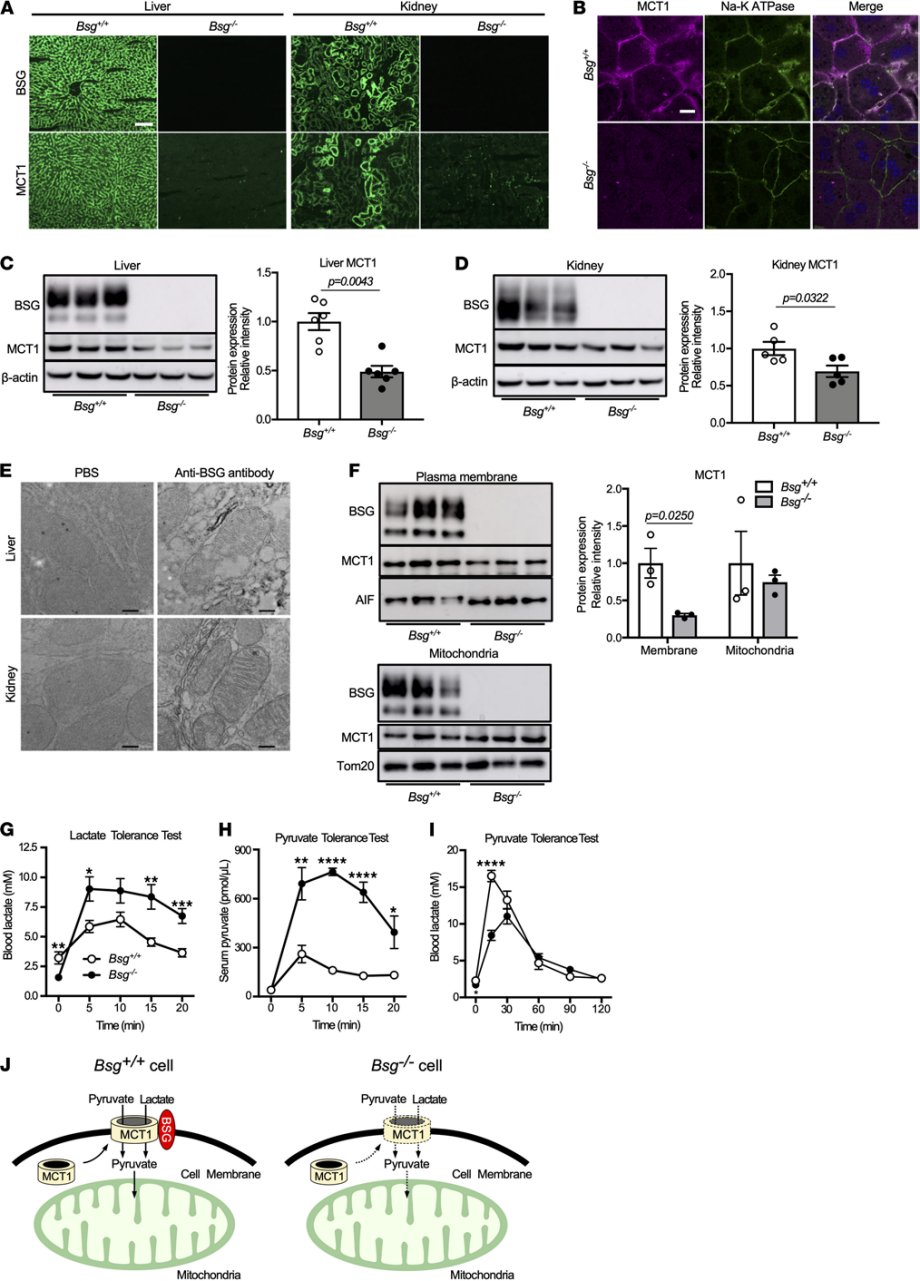
BSG regulates lactate and pyruvate import through interaction with MCTs. (**A**) Immunofluorescence staining for BSG and MCT1 in the liver and renal cortex of tissues from wild-type (*Bsg^+/+^*) and *Bsg*-deficient (*Bsg^–/–^*) mice. Scale bar: 100 μm. (**B**) Representative photographs of isolated *Bsg^+/+^* or *Bsg^–/–^* hepatocytes by immunocytochemical staining. Scale bar: 20 μm. (**C** and **D**) Western blotting analysis of BSG and MCT1 expression in the liver (**C**) and the kidneys (**D**) of *Bsg^+/+^* and *Bsg^–/–^* mice. The intensities of the BSG and MCT1 bands were normalized to those of β-actin, a housekeeping protein that was used as a loading control. White columns and circles, *Bsg^+/+^* mice; gray columns and black circles, *Bsg^–/–^* mice. Data are presented as means ± SEM (*n* = 5–6). Scatter plots display the data for individual mice. (**E**) Immunoelectron microscopic assessment of BSG expression in mitochondria of the liver and kidneys. Scale bar: 200 nm. (**F**) MCT1 expressions in plasma membranes and mitochondria of *Bsg^+/+^* and *Bsg^–/–^* hepatocytes as determined by Western blotting. White columns and circles, *Bsg^+/+^* hepatocytes; gray columns and black circles, *Bsg^–/–^* hepatocytes. Data are presented as means ± SEM (*n* = 3). Scatter plots display the data for individual mice. (**G**) Blood lactate concentrations in *Bsg^+/+^* and *Bsg^–/–^* mice during lactate tolerance tests. (**H** and **I**) Serum pyruvate (**H**) and blood lactate (**I**) concentrations during pyruvate tolerance tests. For **G**–**I**, data are presented as means ± SEM; *n* = 7 (**G**) or 5–9 (**H** and **I**). *P < 0.05, **P < 0.01, ***P < 0.001, ****P < 0.0001, not significant (*P* ≥0.05), for a comparison of *Bsg^+/+^* and *Bsg^–/–^* mice at the indicated time point (2-tailed unpaired Student’s *t* test). (**J**) A schematic illustrating lactate and pyruvate dynamics via MCT-Bsg complexes in hepatocytes derived from *Bsg^+/+^* mice compared with *Bsg^–/–^* hepatocytes.

We first investigated the functional relationship between BSG and MCT1 in the liver and kidney. Both BSG and MCT1 were expressed in the plasma membranes in hepatocytes in the livers of *Bsg^+/+^* mice; similar expression levels were observed in renal tubular epithelial cells (TECs) (Figure 1A). In contrast, MCT1 was expressed at apparently lower levels in hepatocytes and TECs in *Bsg^–/–^* mice. Both MCT1 and MCT4 expressions in skeletal muscles, such as the gastrocnemius and soleus muscles, were also decreased in *Bsg^–/–^* mice (Supplemental Figure 1A; supplemental material available online with this article; https://doi.org/10.1172/jci.insight.142464DS1). *Bsg* deficiency resulted in markedly decreased hepatic MCT1 expression as assessed by immunocytochemical staining and immunoblotting (Figure 1, B and C), indicating that MCT1 failed to accumulate when unable to translocate to the cell surface. The findings in the kidney showed a profile similar to that in the liver (Figure 1D). In addition, immunoelectron microscopy revealed that BSG was present primarily on the inner membrane of mitochondria in the liver and kidney (Figure 1E). Immunoblotting revealed that BSG protein was detectable in the mitochondrial fraction of hepatocytes but was present to a lesser degree in the cytosolic fraction (Supplemental Figure 1B). Interestingly, MCT1 expression in the mitochondria was not affected by *Bsg* deficiency, in contrast to the profile of BSG in the plasma membrane (Figure 1F). The levels of *Slc16a1* mRNA (which encodes MCT1) in the liver and kidneys did not appear to differ in *Bsg^–/–^* mice compared to *Bsg^+/+^* mice (Supplemental Figure 1, C and D). These data collectively suggested that the decreased accumulation of MCT1 in *Bsg^–/–^* animals reflected a plasma membrane–selective effect, rather than a change in the mitochondrial membrane; this change was accomplished at the level of protein translocation, not at that of transcription.

We next examined substrate import in fasted *Bsg^–/–^* mice. Intravenous administration of lactate or pyruvate resulted in significantly larger excursions in the concentrations of circulating lactate or pyruvate in *Bsg^–/–^* mice than in *Bsg^+/+^* mice (Figure 1, G and H). Additionally, fasted *Bsg^–/–^* mice subjected to a pyruvate challenge showed lower blood lactate levels within 30 minutes than were seen in *Bsg^+/+^* mice, indicating a defect in pyruvate uptake due to *Bsg* deficiency, leading in turn to decreased conversion of pyruvate to lactate (Figure 1I). Of note, the blood lactate levels of *Bsg^–/–^* mice at the starting time were lower than those of *Bsg^+/+^* mice, suggesting the impairment of extracellular release of lactate (Figure 1, G and I). We further assessed the involvement of the MCT/BSG axis in terms of the efflux from skeletal muscles using C2C12 cells, a myoblast cell line from adult C3H mouse leg muscles. The inhibition of MCT1 decreased the lactate levels in the media after exposure to a small interfering RNA or the chemical inhibitor AZD3965 (Supplemental Figure 1, E and F). These observations implied impaired import of monocarboxylates in *Bsg^–/–^* mice (Figure 1J).

### Loss of BSG leads to decreased levels of TCA cycle intermediates in the liver and kidneys.

**Figure 2:**
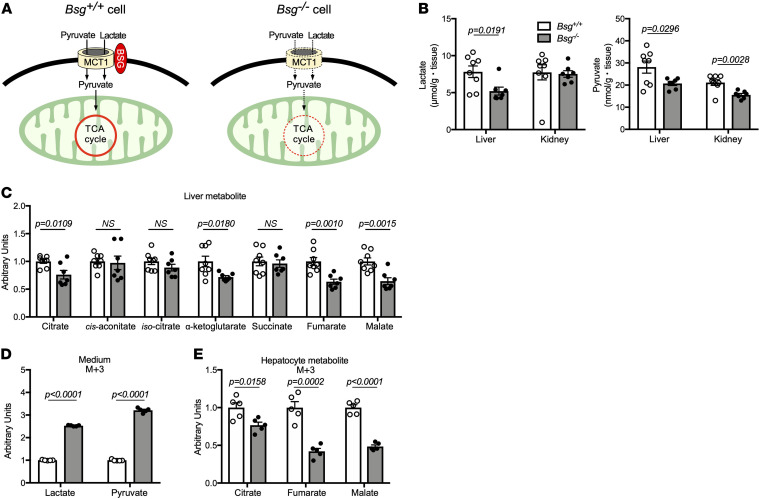
*Bsg* deficiency leads to depletion of TCA cycle intermediates in the liver. (**A**) Schematic illustrating the facilitation by the MCT/BSG complex of the import of substrates used by the TCA cycle. (**B** and **C**) Static metabolome analysis for lactate and pyruvate in livers and kidneys (**B**) and TCA cycle intermediates in livers (**C**) from fasted *Bsg^+/+^* or *Bsg^–/–^* mice. White columns and circles, *Bsg^+/+^* mice; gray columns and black circles, *Bsg^–/–^* mice. Data are presented as means ± SEM (*n* = 7–8). Scatter plots display the data for individual mice. *P* values represent results for a comparison of *Bsg^+/+^* and *Bsg^–/–^* mice for the indicated metabolite (2-tailed unpaired Student’s *t* test). (**D**) Levels of ^13^C_3_-labeled lactate and pyruvate in the media from isolated *Bsg^+/+^* or *Bsg^–/–^* hepatocytes, respectively. White columns, *Bsg^+/+^* hepatocytes; gray columns, *Bsg^–/–^* hepatocytes. *n* = 5 for independent experiments. (**E**) ^13^C_3_-labeled lactate/pyruvate flux into TCA cycle intermediates, such as citrate, fumarate, and malate, in isolated hepatocytes.

Pyruvate and lactate transported into the cells through the MCT/BSG complex are critically linked to the TCA cycle (Figure 2A). Using static metabolome analysis, we further examined the effects of *Bsg* deficiency on the intermediates of the TCA cycle, as well as compensatory changes in other metabolic circuits. *Bsg^–/–^* mice showed lower levels of hepatic lactate and pyruvate, whereas no significant difference in renal lactate values was found, suggesting a compensatory role for MCT2 in the kidneys (Figure 2B). Notably, *Bsg* deficiency led to decreases in the liver levels of TCA cycle intermediates, including citrate, α-ketoglutarate, fumarate, and malate, in fasted mice (Figure 2C). A similar profile was obtained in the kidneys (Supplemental Figure 2A). Since the amounts of TCA intermediates in static metabolomic analysis are measured as a pooled amount in metabolic turnover, we directly evaluated the incorporation of ^13^C_3_-labeled carbon after exposure to ^13^C_3_-labeled lactate/pyruvate in isolated *Bsg^+/+^* or *Bsg^–/–^* hepatocytes, indicating intracellular entry of lactate and pyruvate to the TCA circuit. At 4 hours after exposure, high levels of lactate and pyruvate with ^13^C_3_ enrichment were observed in the media from isolated *Bsg^–/–^* hepatocytes (Figure 2D), suggesting that ^13^C_3_-labeled lactate/pyruvate remained unincorporated in the cells. Notably, ^13^C_3_-labeled TCA cycle intermediates such as citrate, fumarate, and malate were significantly decreased in *Bsg^–/–^* cells (Figure 2E). These data indicated that BSG may promote the TCA cycle in the liver and kidneys through enhanced substrate transport.

### BSG plays a crucial role in glucose production by facilitating intracellular import of lactate and pyruvate.

**Figure 3:**
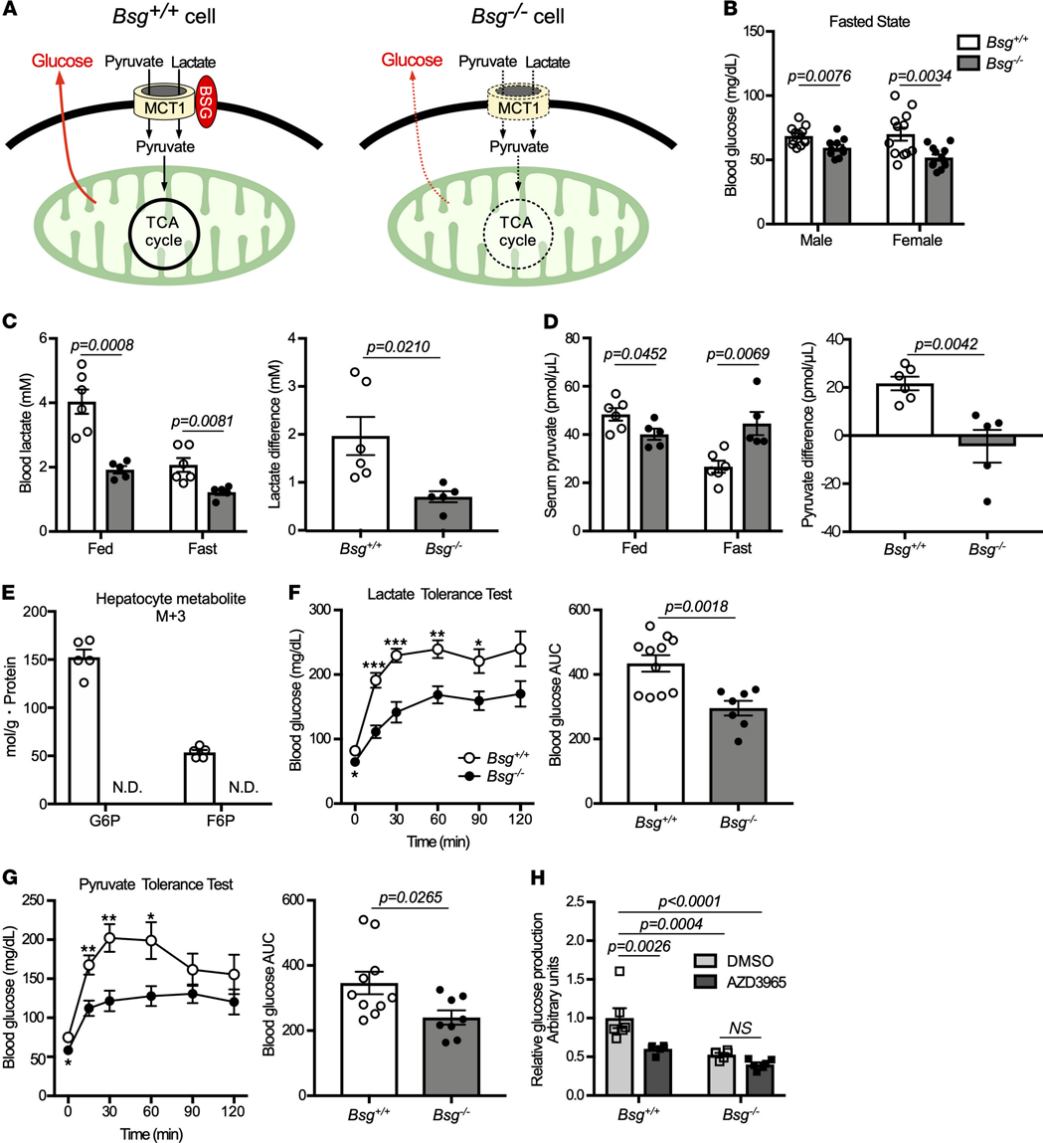
*Bsg* deficiency impairs hepatic availability of lactate and pyruvate in vivo. (**A**) Schematic illustrating impaired gluconeogenesis resulting from decreased substrate import due to *Bsg* deficiency. OAA, oxaloacetate. (**B**) Blood glucose levels in *Bsg^+/+^* and *Bsg^–/–^* mice under fasting conditions. White columns and circles, *Bsg^+/+^* mice; gray columns and black circles, *Bsg^–/–^* mice (*n* = 10–12/genotype). Scatter plots display the data for individual mice. (**C**) Differences in blood lactate values between feeding and fasting states in *Bsg^+/+^* or *Bsg^–/–^* female mice (*n* = 5–6/genotype). (**D**) Differences in serum pyruvate values between feeding and fasting states in *Bsg^+/+^* or *Bsg^–/–^* female mice (*n* = 5–6/genotype). (**E**) G6P and F6P with incorporation of ^13^C_3_-labeled carbon in isolated *Bsg^+/+^* or *Bsg^–/–^* hepatocytes. White columns, *Bsg^+/+^* hepatocytes; gray columns, *Bsg^–/–^* hepatocytes. *n* = 5 for independent experiments. N.D., no detection. (**F**) Blood glucose excursions and the AUC scores in fasting *Bsg^+/+^* and *Bsg^–/–^* female mice during lactate tolerance tests (*n* = 7–11/genotype). (**G**) Blood glucose excursions and AUC scores during pyruvate tolerance tests in female mice (*n* = 8–10/genotype). (**H**) Endogenous glucose production in isolated hepatocytes of *Bsg^+/+^* and *Bsg^–/–^* female mice cultured in medium supplemented with 20 mM sodium lactate and 2 mM sodium pyruvate in the absence or presence of 100 nM AZD3965 (an inhibitor of MCT1 activity). *n* = 6 for independent experiments. For all relevant panels, data are presented as means ± SEM. *P < 0.05, **P < 0.01, ***P < 0.001, for the comparison of *Bsg^+/+^* and *Bsg^–/–^* at the indicated time point (2-tailed unpaired Student’s *t* test).

Lactate and pyruvate transported into cells by MCT/BSG complexes are a major source of gluconeogenesis in mammals (Figure 3A). Impairment of this substrate import results in a decrease in glucose production. We further addressed the role of MCT/BSG complexes in gluconeogenesis by comparing metabolic profiles in *Bsg^+/+^* and *Bsg^–/–^* mice. In both males and females, fasting blood glucose levels in *Bsg^–/–^* mice were lower than those in *Bsg^+/+^* mice (Figure 3B). The circulating levels of both lactate and pyruvate fell by over 50% in fasted *Bsg^+/+^* mice compared with fed *Bsg^+/+^* mice; smaller differences were observed for lactate and pyruvate values when comparing between fed and fasted conditions in *Bsg^–/–^* mice (Figure 3, C and D). Additional in vitro studies with stable isotope ^13^C_3_-labeled lactate/pyruvate in isolated *Bsg^+/+^* or *Bsg^–/–^* hepatocytes were further conducted to evaluate the involvement of BSG in the process of gluconeogenesis. In the absence of glucose, glucose 6-phosphate (G6P) and fructose 6-phosphate (F6P) with ^13^C_3_ incorporation, which are included in the rate-limiting step of gluconeogenesis, could hardly be found in isolated *Bsg^–/–^* hepatocytes at 4 hours after exposure (Figure 3E). These data suggested decreased intracellular utilization of lactate and pyruvate in *Bsg^–/–^* mice compared with *Bsg^+/+^* animals. Consistent with this idea, blood glucose levels in *Bsg^–/–^* mice appeared to be suppressed in lactate tolerance tests in fasted animals (Figure 3F). The profile in the pyruvate tolerance test was in agreement with that in the lactate tolerance test (Figure 3G). In contrast, mRNA levels in the liver and kidneys for genes encoding major gluconeogenic enzymes, including pyruvate carboxylase, phosphoenolpyruvate carboxykinase 1, and glucose-6-phosphatase, were not decreased in fasted *Bsg^–/–^* mice (Supplemental Figure 3, A and B). Thus, *Bsg* deficiency did not directly affect the expression of genes encoding gluconeogenic enzymes. We further examined whether endogenous glucose production depends on MCT1 expression using primary cultured hepatocytes derived from *Bsg^+/+^* or *Bsg^–/–^* mice. Notably, MCT1 inhibition by a chemical reagent (AZD3965) led to a decrease in glucose production by primary cultured hepatocytes from *Bsg^+/+^* mice, such that the glucose level fell to a level similar to that in hepatocytes from *Bsg^–/–^* mice when the cells were grown without the inhibitor (Figure 3H). These data suggested that BSG might contribute to gluconeogenesis by increasing the intracellular availability of lactate and pyruvate through MCT1.

### Bsg^–/–^ mice compensate for impaired gluconeogenesis by promoting the utilization of glucogenic amino acids.

**Figure 4:**
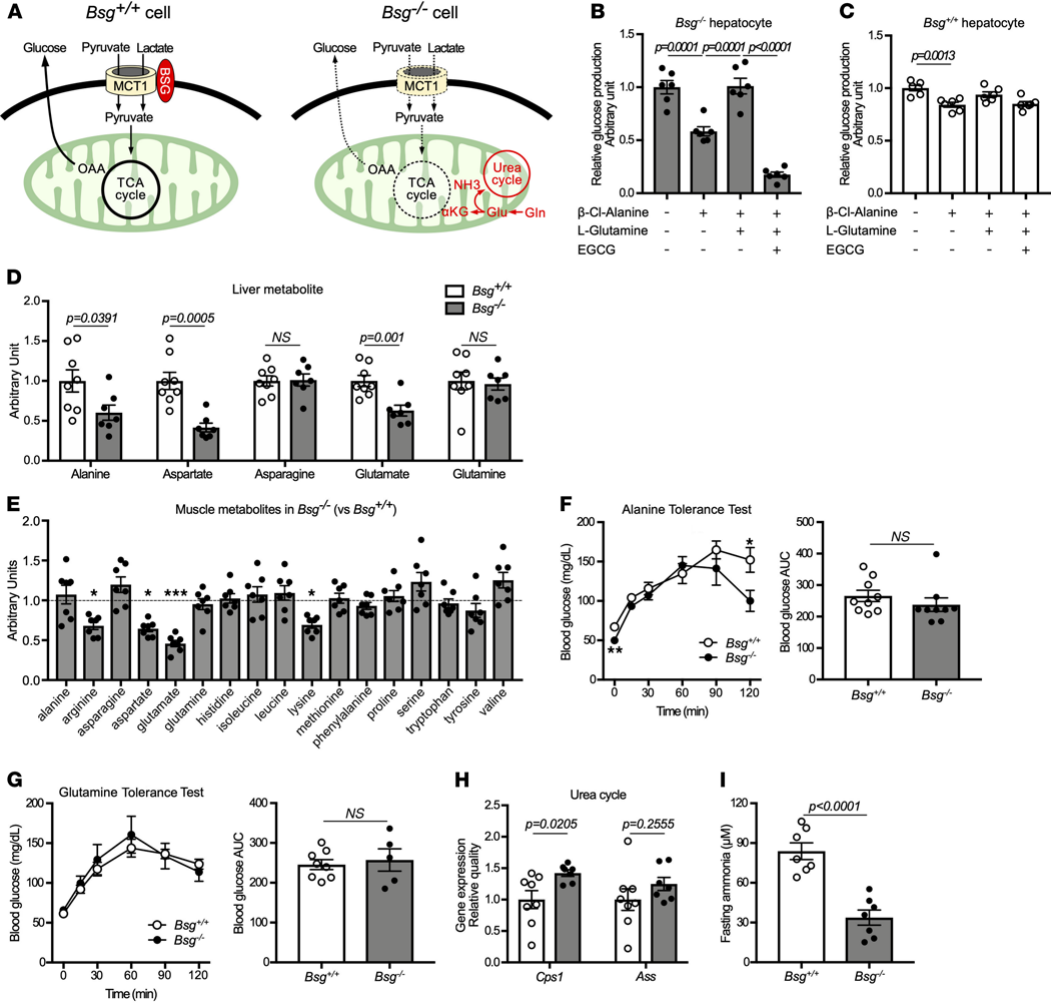
*Bsg* deficiency is compensated for by alternative circuits that provide utilization of glycogenic amino acids into the TCA cycle. (**A**) Schematic illustrating use of the glycogenic amino acid pathway to compensate for impaired gluconeogenesis resulting from *Bsg* deficiency. Ala, alanine; NH3, ammonia; αKG, α-ketoglutaric acid; Glu, glutamate; Gln, glutamine. (**B** and **C**) Endogenous glucose production in hepatocytes isolated from *Bsg^–/–^* (**B**) and *Bsg^+/+^* (**C**) animals when cells were cultured in the absence or presence of 250 μM β-chloro-l-alanine hydrochloride (an inhibitor of Ala transaminase), 10 mM l-glutamine, and 20 μM EGCG (an inhibitor of Glu dehydrogenase) for 4 hours. Gray columns and black circles, *Bsg^–/–^*; white columns and circles, *Bsg^+/+^* (*n* = 6/genotype). Scatter plots display the data for individual mice. Statistical analyses were performed using a 1-way ANOVA with post hoc Tukey’s test for multiple comparisons. (**D** and **E**) Static metabolome analysis of amino acids in livers (**D**) and skeletal muscles (**E**) from fasted *Bsg^+/+^* or *Bsg^–/–^* mice (*n* = 7–8/genotype). Levels of amino acids in *Bsg^–/–^* skeletal muscles are exhibited with respect to those of *Bsg^+/+^* muscles. (**F** and **G**) Blood glucose levels and AUC scores in fasting *Bsg^+/+^* and *Bsg^–/–^* mice during alanine (**F**) or glutamine (**G**) tolerance tests (*n* = 5–9/genotype). (**H**) Expression levels of transcripts encoding ureagenesis-related proteins, including CPS1 and argininosuccinate synthase (ASS). mRNA levels were normalized to those encoding the housekeeping protein β-actin (*n* = 7–8/genotype). (**I**) Serum ammonia values in fasting *Bsg^+/+^* and *Bsg^–/–^* mice (*n* = 7/genotype). For all relevant panels, data are presented as means ± SEM. *P < 0.05, **P < 0.01, ***P < 0.001, for the comparison of *Bsg^+/+^* and *Bsg^–/–^* at the indicated time point (2-tailed unpaired Student’s *t* test).

Instead of being blocked in intracellular transport of monocarboxylates, *Bsg^–/–^* mice may employ a compensatory mechanism (Figure 4A). Gluconeogenesis can be mediated through bidirectional cycling of the alanine/pyruvate axis via catalysis by alanine transaminase (or alanine aminotransferase, ALT) and adaptive redirection to the TCA cycle; this alternative pathway may predominate under conditions of diminished pyruvate availability (22, 23). Glutamine also may contribute to gluconeogenesis through its conversion to glutamate and subsequently to α-ketoglutaric acid, reactions that are mediated by glutaminase and glutamate dehydrogenase, respectively (24). To assess how the utilization of alanine and glutamine compensates for decreased mitochondrial lactate and pyruvate import, primary cultures of hepatocytes derived from *Bsg^+/+^* or *Bsg^–/–^* mice were tested for their ability to produce glucose when incubated with lactate and pyruvate in conjunction with the inhibition of ALT by β-Cl-alanine and of glutamate dehydrogenase by epigallocatechin 3-gallate (EGCG). Notably, glucose production was significantly suppressed in ALT-inhibited *Bsg^–/–^* hepatocytes in the presence of β-Cl-alanine (Figure 4B). Under conditions that prevented alanine utilization, l-glutamine administration restored glucose production by *Bsg^–/–^* hepatocytes to its original levels. In contrast, a marked decrease in glucose production was found in *Bsg^–/–^* hepatocytes treated with both β-Cl-alanine and EGCG. These inhibitors (alone or together) had little effect on *Bsg^+/+^* hepatocytes (Figure 4C). Indeed, data obtained in an in vivo static metabolome analysis revealed an apparent decrease in the levels of amino acids following gluconeogenesis in the liver and skeletal muscles of fasted *Bsg^–/–^* mice (Figure 4, D and E). The profile of amino acids in the kidneys of these animals was similar to that in the liver (Supplemental Figure 4A). Interestingly, glucogenic amino acids, such as alanine, aspartate, and glutamate, which were apparently reduced in the livers of fasted *Bsg^–/–^* mice, showed no significant differences in serum amino acids between *Bsg^+/+^* and *Bsg^–/–^* mice (Figure 4D, Supplemental Figure 4B). However, asparagine and glutamine, which exhibited no obvious decreases in *Bsg^–/–^* livers, were significantly increased in *Bsg^–/–^* sera. Furthermore, dosing of *Bsg^–/–^* mice with alanine produced a glucose excursion similar to that seen in *Bsg^+/+^* mice (Figure 4F). Glucose excursion of *Bsg^–/–^* mice after glutamine treatment was similar to that seen in *Bsg^+/+^* mice (Figure 4G). As nitrogen metabolites derived from glycogenic amino acids are processed in the urea cycle, we next examined ureagenesis. While the levels of glucogenic amino acids (including alanine, aspartate, and glutamate) were decreased in the *Bsg^–/–^* liver (Figure 4D), *Bsg^–/–^* mice exhibited increases in the liver levels of urea cycle intermediates, including arginine, citrulline, ornithine, and creatinine (Supplemental Figure 4C). The levels of transcripts of genes encoding proteins essential for ureagenesis (such as carbamoyl phosphate synthase [CPS] 1) were increased in the livers of fasted *Bsg^–/–^* mice compared with *Bsg^+/+^* mice (Figure 4H). Serum ammonia levels in *Bsg^–/–^* mice also were suppressed under fasting conditions (Figure 4I). These findings suggested that *Bsg* deficiency can be circumvented by compensatory activation of pyruvate-alanine cycling and redirection of flux from glutamine to the TCA cycle.

### Ketogenesis arising from fatty acid β-oxidation is enhanced in Bsg^–/–^ mice.

**Figure 5:**
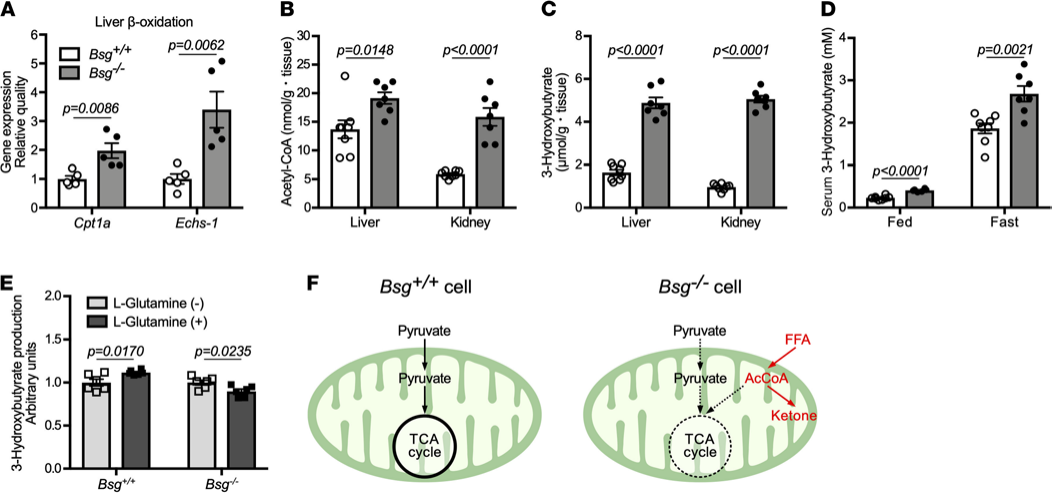
Increased ketogenesis via activation of fatty acid β-oxidation in *Bsg* deficiency. (**A**) Concentrations of mRNAs encoding CPT1a and ECHS1 proteins, both of which are involved in fatty acid β-oxidation in the liver under fasting conditions (*n* = 5/genotype). mRNA levels were normalized to those encoding the housekeeping protein β-actin. White columns and circles, *Bsg^+/+^* mice; gray columns and black circles, *Bsg^–/–^* mice. Scatter plots display the data for individual mice. (**B** and **C**) AcCoA (**B**) and the ketone body 3-hydroxybutyrate (**C**) contents in the livers and kidneys in fasting *Bsg^+/+^* and *Bsg^–/–^* mice (*n* = 7–8/genotype). (**D**) Serum 3-hydroxybutyrate values in *Bsg^+/+^* and *Bsg^–/–^* mice under feeding and fasting conditions (*n* = 6–8/genotype and condition combination). (**E**) 3-Hydroxybutyrate production by isolated hepatocytes derived from *Bsg^+/+^* and *Bsg^–/–^* mice when the cells were cultured in the presence of l-glutamine (*n* = 6/genotype). For all relevant panels, data are presented as means ± SEM. For the comparison of *Bsg^+/+^* and *Bsg^–/–^*, we used 2-tailed unpaired Student’s *t* test. (**F**) Schematic illustrating increased lipolysis and ketogenesis in *Bsg*-deficient cells. FFA, free fatty acid; AcCoA, acetyl-CoA.

In fasted *Bsg^–/–^* mice, the TCA cycle was suppressed, and the urea cycle was activated to dispose of the ammonia that was generated by the utilization flux of glucogenic amino acids (e.g., alanine and glutamine) into the TCA cycle (Figure 4, H and I, and Supplemental Figure 4C). A series of metabolic circuits that connect β-oxidation of fatty acids to ketogenesis was further examined. *Bsg^–/–^* mice showed increases in the transcripts encoding palmitoyltransferase-1a (CPT1a) and enoyl-CoA hydratase (ECHS1), mitochondrial enzymes involved in fatty acid β-oxidation in the liver under fasting conditions (Figure 5A). Consistent with this, fasted *Bsg^–/–^* mice exhibited higher levels of acetyl-CoA and 3-hydroxybutyrate (a ketone body) in the liver and kidney (Figure 5, B and C). Likewise, serum 3-hydroxybutyrate concentrations were significantly higher in *Bsg^–/–^* mice than in *Bsg^+/+^* mice under both feeding and fasting conditions (Figure 5D). If anaplerosis is partly compensated by increasing the supply of a TCA cycle intermediate (e.g., α-ketoglutaric acid through l-glutamine administration), the incorporation of acetyl-CoA, a β-oxidation product, into the TCA cycle would be enhanced and thus reduce ketone production. Consistent with this idea, l-glutamine treatment of *Bsg^–/–^* hepatocytes yielded decreased 3-hydroxybutyrate levels (Figure 5E). Thus, ketogenesis may be negatively associated with anaplerosis of the TCA cycle. Collectively, our data support the idea that a decrease in anaplerosis interrupts the flux of acetyl-CoA into the TCA cycle from β-oxidation of fatty acids, thereby activating ketone body synthesis using acetyl-CoA (Figure 5F) (5, 25).

### Bsg^–/–^ mice are protected from HFD-induced insulin resistance.

**Figure 6:**
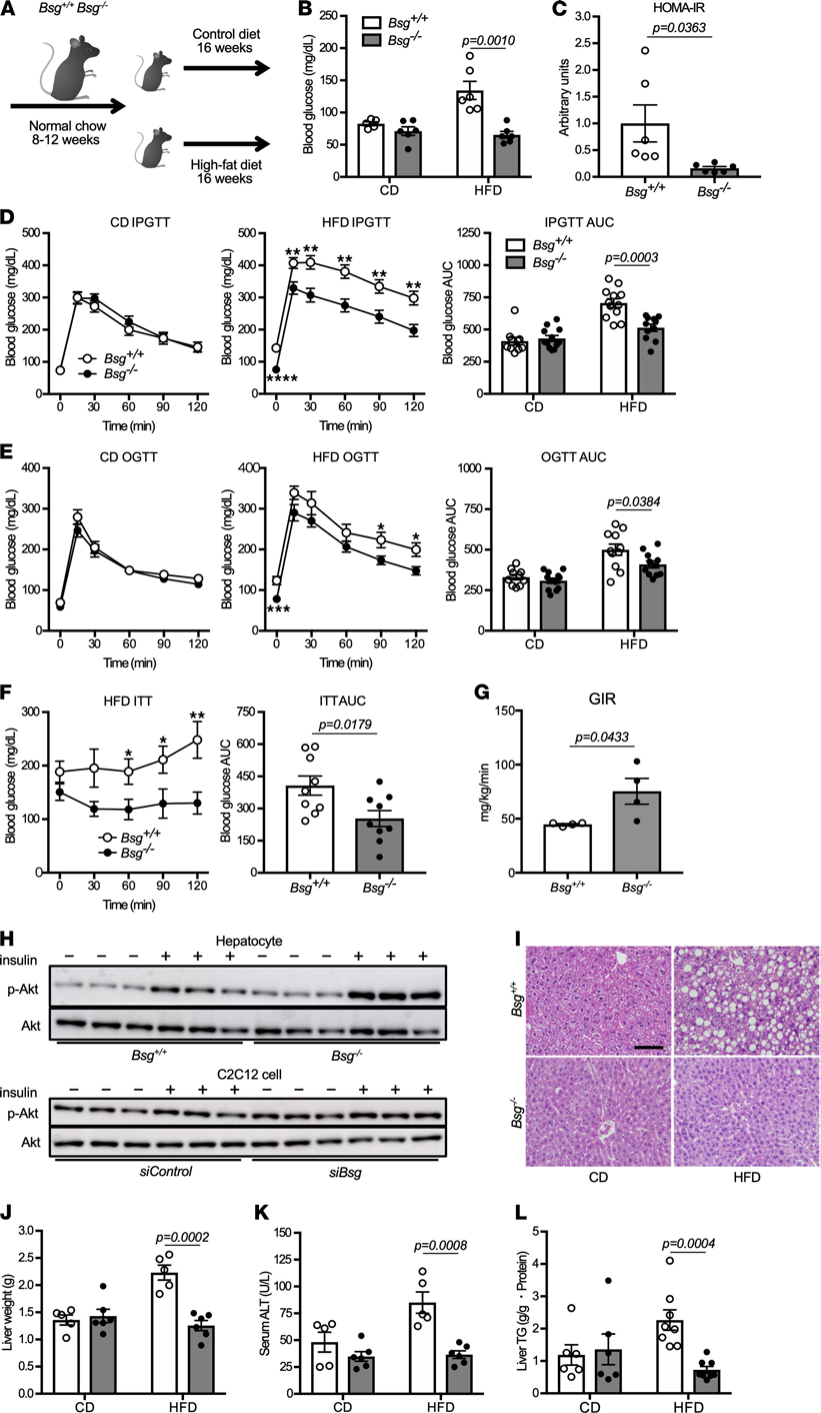
*Bsg* deficiency protects mice from HFD-induced insulin resistance. (**A**) Schematic diagram of the in vivo experiments used to test the effects of 16 weeks of nutritional overburden. (**B**) Fasting blood glucose levels in *Bsg^+/+^* and *Bsg^–/–^* mice maintained for 16 weeks on standard CD or HFD (*n* = 5–6/genotype and diet combination). White columns and circles, *Bsg^+/+^* mice; gray columns and black circles, *Bsg^–/–^* mice. Scatter plots display the data for individual mice. (**C**) HOMA-IR in HFD-fed *Bsg^+/+^* and *Bsg^–/–^* mice (*n* = 6/genotype). (**D** and **E**) Glucose excursions and the AUC scores in *Bsg^+/+^* and *Bsg^–/–^* mice (maintained on CD or HFD for 16 weeks) when subjected to (**D**) IP glucose tolerance tests or (**E**) oral glucose tolerance tests (*n* = 10–12/genotype and diet combination). (**F**) Glucose excursions and the AUC scores in *Bsg^+/+^* or *Bsg^–/–^* mice (maintained on HFD for 16 weeks) when administered IP with insulin (0.6 units/kg body weight) (*n* = 9/genotype). (**G**) GIR in 8- to 12-week-old *Bsg^+/+^* or *Bsg^–/–^* mice after insulin infusion in the hyperinsulinemic-euglycemic clamp study (*n* = 4/genotype). (**H**) Akt phosphorylation in isolated hepatocytes and C2C12 cells in the absence or presence of insulin as demonstrated by Western blotting analysis. (**I**) Representative micrographs of H&E-stained sections showing hepatic steatosis in *Bsg^+/+^* and *Bsg^–/–^* mice. Scale bar: 200 μm. (**J**–**L**) Liver weight (**J**), serum ALT values (**K**), and liver TG content (**L**) in CD-fed or HFD-fed *Bsg^+/+^* and *Bsg^–/–^* mice (*n* = 5–8/genotype and diet combination). For all relevant panels, data are presented as means ± SEM. *P < 0.05, **P < 0.01, for the comparison of *Bsg^+/+^* and *Bsg^–/–^* at the indicated time point (2-tailed unpaired Student’s *t* test).

Accumulation of fatty acids and triglycerides alters mitochondrial function, causing an impaired capacity of insulin to suppress hepatic gluconeogenesis in fatty liver, leading in turn to the development of insulin resistance (5, 8, 26). To elucidate the role of BSG in the development of glucose intolerance, *Bsg^+/+^* and *Bsg^–/–^* mice were fed a 60% fat diet (HFD) or a 10% fat diet (standard chow diet, CD) during a 16-week experimental interval (Figure 6A). When maintained on HFD, both genotypes showed a gradual increase in body weight during the study period compared with CD-fed mice of the respective genotypes. With either diet, the body weights of the *Bsg^–/–^* mice were nominally lower than those of the *Bsg^+/+^* mice during the experimental period, but these differences fell short of significance (Supplemental Figure 5A). With either HFD or CD, animals of the 2 genotypes consumed amounts of food corresponding to similar total energy content (Supplemental Figure 5B).

After 16 weeks, the HFD-fed *Bsg^+/+^* mice had elevated fasting blood glucose levels compared with HFD-fed *Bsg^–/–^* mice; in addition, HFD- and CD-fed *Bsg^–/–^* mice had similar fasting blood glucose levels (Figure 6B). The homeostatic model assessment of insulin resistance (HOMA-IR) was significantly decreased in HFD-fed *Bsg^–/–^* mice compared with HFD-fed *Bsg^+/+^* mice (Figure 6C). Interestingly, the magnitude of the elevation in glucose levels in HFD-fed *Bsg^–/–^* mice was significantly lower than that in HFD-fed *Bsg^+/+^* mice during intraperitoneal (IP) and oral glucose tolerance tests (Figure 6, D and E). AUCs in both tolerance tests also were decreased in HFD-fed *Bsg^–/–^* mice compared with HFD-fed *Bsg^+/+^* mice. The curves of blood glucose levels in *Bsg^+/+^* mice appeared to be similar to those in *Bsg^–/–^* mice. In contrast, CD-fed *Bsg^+/+^* and *Bsg^–/–^* mice exhibited profiles similar to each other in each of the glucose tolerance tests. Notably, the IP administration of insulin (0.6 units/kg body weight) to *Bsg^+/+^* mice maintained for 16 weeks on HFD did not suppress fasting blood glucose levels for 120 minutes, unlike the case in insulin-dosed HFD-fed *Bsg^–/–^* mice (Figure 6F). We performed a hyperinsulinemic-euglycemic clamp technique to further assess the effect of BSG on insulin sensitivity. Similar to the profile of the insulin tolerance test, the glucose infusion rate (GIR) in *Bsg^–/–^* mice was strikingly higher than that in *Bsg^+/+^* mice (Figure 6G). In vitro studies with isolated hepatocytes and myoblastic C2C12 cells were performed to validate insulin signaling in the liver. Phosphorylation of Akt, which is negatively correlated with insulin resistance, was markedly increased under *Bsg* deficiency (Figure 6H). These observations indicated that *Bsg* deficiency prevents the impaired glucose homeostasis otherwise observed in HFD-fed mice. To clarify the *Bsg* deficiency–associated suppression of gluconeogenesis that otherwise would be induced in response to chronic nutritional overburden, lactate and pyruvate tolerance tests were performed in the HFD- and CD-fed wild-type and mutant mice. In both tolerance tests, higher values of blood glucose were observed in HFD-fed *Bsg^+/+^* mice than in HFD-fed *Bsg^–/–^* mice (Supplemental Figure 5, C and D). For HFD-fed mice of either genotype, the glucose excursions induced by lactate were of similar magnitudes to those seen following treatment with pyruvate. These data demonstrated that *Bsg^–/–^* mice are protected from the impaired capacity of insulin to suppress gluconeogenesis otherwise caused by maintenance on the HFD.

To determine the mechanism whereby *Bsg^–/–^* mice are rendered resistant to HFD-induced hepatic steatosis, pathological and immunochemical analyses were performed. Histological findings demonstrated that HFD-fed *Bsg^+/+^* mice exhibited severe steatosis, which can be observed in nonalcoholic fatty liver disease (NAFLD), but these findings were decreased in HFD-fed *Bsg^–/–^* mice (Figure 6I). Consistent with these observations, significant increases in liver weight, liver triglyceride (TG) content, and serum ALT levels were found in HFD-fed *Bsg^+/+^* mice compared with HFD-fed *Bsg^–/–^* mice (Figure 6, J–L). Indeed, the values of these parameters in HFD-fed *Bsg^–/–^* mice remained similar to those observed in CD-fed *Bsg^–/–^* mice. Since ketone bodies produced by the oxidation of free fatty acids are an alternative metabolic substrate during starvation, 3-hydroxybutyrate levels in the liver and kidney were quantified in mice with HFD-induced obesity. Despite the lack of a significant difference in visceral fat content and serum TG values (Supplemental Figure 5E), HFD-fed *Bsg^–/–^* mice possessed significantly higher liver and kidney levels of 3-hydroxybutyrate than HFD-fed *Bsg^+/+^* mice (Supplemental Figure 5F). The respiratory quotient was further evaluated in HFD-fed mice to clarify the utilization of fat as a respiratory substrate. Notably, no significant differences in respiratory quotient between *Bsg^+/+^* and *Bsg^–/–^* mice with HFD loading were observed (Supplemental Figure 5G). *Bsg* deficiency has several effects on the energy circuits in hepatocytes but does not necessarily result in significant differences in the respiratory quotients and volume of oxygen consumption in vivo. Recent investigations have documented the beneficial effects of ketone bodies against inflammation and oxidative stress (5, 27, 28). Consistent with those biochemical and pathological findings, the levels of transcripts encoding proteins involved in the response to oxidative stress, e.g., NF-E2-related factor-2, were significantly increased in the livers of HFD-fed *Bsg^+/+^* mice compared with those in HFD-fed *Bsg^–/–^* mice (Supplemental Figure 5H). These data demonstrated that *Bsg* deficiency ameliorates NAFLD caused by oxidative stress in HFD-fed mice.

## Discussion

Despite the importance of lactate and pyruvate import in metabolic homeostasis, relatively little is understood about the metabolic impacts of the MCT/BSG complex. The current study demonstrates that the MCT/BSG complex is indeed positioned at a crucial intersection of cellular metabolism. Thus, *Bsg* deficiency reduced the entry of lactate and pyruvate into cells and consequently suppressed the TCA cycle and gluconeogenesis. This was supported by the finding that glucogenic amino acids (alanine and glutamine) partially compensated for the decreased gluconeogenesis in *Bsg*^–/–^ mice. The enhanced production of ketone bodies found in these mice is also consistent with this. This net anaplerosis suppression compensatively elicited the utilization of glucogenic amino acids and activation of ketone body production to maintain intracellular homeostasis. *Bsg* deficiency might cause the increased consumption of glucogenic amino acids, including glutamate and aspartate, in skeletal muscles resulting from a potential shift to catabolic status.

The import of substrates responsible for gluconeogenesis and the TCA cycle into the mitochondria presumably must be coordinated via various import shuttles that include MCT/BSG complexes and the mitochondrial pyruvate carrier (MPC) (22, 29–31). The present study suggested that *Bsg* deficiency may not affect MCT1 expression in mitochondria. In other words, the effect of BSG on MCT1 localization might be plasma membrane selective. Indeed, it is conceivable that loss of mitochondrial pyruvate transport might be caused by *Bsg* deficiency despite the presence of MCT1 in the mitochondria. However, pyruvate uptake by the cell was suppressed in *Bsg*^–/–^ mice (Figure 1H), and glycolysis was suppressed in *Bsg*^–/–^ hepatocytes in our Seahorse study (data not shown) and a previous study (32). Therefore, tracer metabolomics using ^13^C_3_-labeled glucose or pyruvate might not precisely assess mitochondrial pyruvate transport in *Bsg*^–/–^ hepatocytes, and this issue is an important topic of future investigation. Interpretation of this finding is important to understanding the whole picture of the transport of lactate and pyruvate. Both compounds are shuttled through MCTs in the plasma membrane as well as those in the mitochondrial inner membrane, although the functions of mitochondrial MCTs remain elusive. Lactate dehydrogenase catalyzes the reversible oxidation of lactate to pyruvate, which may occur in both the cytosol and mitochondria, thereby providing pyruvate to the TCA cycle. Indeed, Brooks and colleagues demonstrated that the MCT/BSG complex is associated with lactate dehydrogenase in the mitochondria (31, 33, 34). Mitochondrial pyruvate also serves as an important precursor in gluconeogenesis; given that pyruvate cannot be directly fluxed to the cytosol, the compound is converted to oxaloacetate and then to phosphoenolpyruvate, which subsequently is transferred to the cytosol, where gluconeogenesis is completed. However, cytosolic pyruvate is also transported into the mitochondria through MPC. The functions of MCT1 and MPC1/2 on the mitochondrial inner membrane may overlap (at least in part) in terms of pyruvate transportation. In the present work, we showed that *Bsg^–/–^* mice exhibited impaired gluconeogenesis due to decreased entry of lactate and pyruvate into the cell even though mitochondrial MCT1 and MPC1/2 might be intact. Interestingly, a similar phenotype has been observed in hepatic *Mpc2*-deficient mice (22).

The plasma membrane–selective abolishment of MCT1 by *Bsg* depletion may provide a chance to modulate cellular metabolism to treat human diseases. BSG inhibition might be better than MCT1 inhibition, as the latter shuts down both plasma and mitochondrial MCT1, and exhibits more severe phenotypes, such as stronger attenuation of lactate entry into the cell, than the former (35). In the present study, we found that BSG inhibition had the potential to ameliorate insulin resistance and hepatic steatosis. Other investigators demonstrated that CD147/BSG is associated with hepatic steatosis and autophagy in both patients with NAFLD and liver-specific *Bsg^–/–^* mice (36). The differences between parts of the results of these 2 studies may be due to the presence or absence of substrate transport between multiple organs. Our in vivo study demonstrated that *Bsg^–/–^* mice are resistant to HFD-induced diabetic hyperglycemia; excessive gluconeogenesis and diabetes mellitus with insulin resistance were suppressed by *Bsg* deficiency. Furthermore, *Bsg* deficiency significantly improved hepatic insulin signaling in mice with diet-induced obesity, as evidenced by several in vivo tolerance tests and in vitro biochemical analyses. Consistent with our findings, *Mct1*-haploinsufficient mice do not develop the hepatic steatosis that is induced by activating mutations in AMPK; this resistance is mediated by decreased lactate metabolism (37). Ketone bodies and lactate mitigate the tissue damage the release of reactive oxygen species causes (38). Hence, it was assumed that the degeneration of cell surface MCTs may be characterized by deterioration of the liver and kidneys of *Bsg^–/–^* mice. In an elegant study by Ruegsegger and colleagues (39), indeed, MCTs’ inhibition under insulin deprivation disrupts mitochondrial homeostasis in the brain. When accompanied by the suppressed lactate import to the liver and kidneys of *Bsg^–/–^* mice, ketone synthesis from free fatty acids’ β-oxidation is strikingly activated in *Bsg* deficiency. Because ketone bodies suppress hepatic steatosis (5, 40, 41), the elevation of ketone production in *Bsg^–/–^* mice may contribute, at least in part, to the amelioration of hepatic steatosis. This amelioration may also be partly due to the attenuation of oxidative stress as a result of the enhanced ketone synthesis. The involvement of BSG in the antioxidant protection of various organs will require further investigation. Taken together, our results suggest that an in-depth understanding of BSG-associated metabolic circuits will be of use in developing and refining therapeutics for diabetes and related diseases.

## Methods

### Animals and experimental design.

BSG is an important cell surface molecule involved in early embryogenesis and reproduction (42). Because mice deficient in the *Bsg* gene (*Bsg^–/–^*) are rarely born through ordinary mating, we established the following protocol (43). Briefly, *Bsg*^+/–^ mice with 129/SV background were backcrossed with C57BL/6J mice to produce F1 hybrid offspring (“reverse F1 hybrid”). By intercrossing these mice, mixed “reverse F2” were generated and were used in this study. All experiments were performed with *Bsg*^+/+^ and *Bsg*^–/–^ littermates. The mice used were 8- to 12-week-old males and females; the mice were housed under specific pathogen–free conditions in temperature- and humidity-controlled rooms maintained on a 12-hour dark/12-hour light cycle. Mice were provided with ad libitum access to tap water and the respective preassigned chow, except as indicated for food fasting. For HFD studies, male mice weighing 20–30 g each were provided with ad libitum access to either CD chow consisting of 10% calories from fat (12450J, Research Diets, Inc.) or HFD chow consisting of 60% calories from fat (D12492, Research Diets, Inc.) for a 16-week study interval. The mice then were subjected to glucose, pyruvate, and lactate tolerance studies as indicated below. At the study end, each animal was euthanized following (as indicated) terminal blood collection, and the liver, kidneys, and skeletal muscles, such as gastrocnemius and soleus muscles, then were recovered.

### Liver and kidney histology.

For standard histopathology, liver and kidney tissues were incubated in 10% formalin, embedded in paraffin, sectioned at 1 μm thickness, transferred to glass slides, and stained with H&E by standard methodologies. For immunohistochemistry, separate segments of the tissues were embedded in OCT compound (Leica Microsystems) and frozen in liquid nitrogen. A cryotome (Leica Microsystems) was used to generate 4 μm thick sections that then were transferred to slides and processed by standard methodologies. The resulting sections were stained with rabbit monoclonal anti–mouse BSG antibody (Ab) (catalog ab212057; Abcam), rabbit anti–mouse MCT1 Ab (catalog 20139-1-AP; Proteintech), rabbit anti–mouse MCT4 Ab (catalog 22787-1-AP; Proteintech), or FITC-conjugated goat anti–mouse IgG Ab (catalog 115-095-062; Jackson ImmunoResearch).

### Cell culture.

Mouse hepatocyte primary cultures were established from the livers of adult *Bsg*^+/+^ and *Bsg*^–/–^ mice according to the previously described method (44). In short, immediately following euthanasia of the animals, livers were perfused sequentially with buffered Liver Perfusion Medium (catalog 17701-038; Thermo Fisher Scientific) and Liver Digest Medium (catalog 17703-034; Thermo Fisher Scientific). Hepatocytes from the removed livers were extracted into William’s E Medium (catalog A1217601; Thermo Fisher Scientific) containing Primary Hepatocyte Thawing and Plating Supplements (catalog CM3000; Thermo Fisher Scientific). Primary cultured hepatocytes were maintained in William’s E Medium supplemented with Maintenance Supplements (catalog CM4000; Thermo Fisher Scientific).

### Immunocytochemistry.

After plating of hepatocytes extracted from *Bsg^+/+^* or *Bsg*^–/–^ mice, preparation for immunocytochemistry was performed as described previously (45). In brief, samples were fixed with ice-cold ethanol, incubated with a blocking solution containing 5% normal goat serum (catalog S-1000; Vector Laboratories) and 0.1% Triton X-100 (MilliporeSigma), and washed twice with phosphate-buffered saline (PBS). Hepatocytes were stained with rabbit anti–mouse MCT1 Ab (catalog 20139-1-AP; Proteintech) or mouse monoclonal anti–sodium/potassium ATPase Ab (catalog Ab7671; Abcam) and diluted in PBS containing 2.5% normal goat serum, followed by secondary hybridization with Alexa Fluor 488–conjugated goat anti–mouse IgG Ab (catalog A32723; Thermo Fisher Scientific) or Alexa Fluor 555–conjugated goat anti–rabbit IgG Ab (catalog A32732; Thermo Fisher Scientific), respectively. Cells then were incubated with DAPI solution (catalog 340-07971; Dojindo Laboratories) and embedded with FluorSave reagent (catalog 345789; MilliporeSigma). Images were acquired using a TiEA1R microscope (Nikon, Inc., Tokyo, Japan) equipped with a Plan Apo λ ×100 numerical aperture 1.45 oil immersion objective lens (Nikon, Inc.) and a GaAsP detector (Nikon, Inc.) in a fixed image acquisition setting. The fluorescence intensities of the resulting images were analyzed with NIS-Elements.

### Western blot analysis.

Mouse liver and kidney tissues, as well as hepatocytes cultured in vitro, were lysed in RIPA buffer (catalog sc-24948; Santa Cruz Biotechnology). Western blotting was conducted as described previously (46). Briefly, after proteins were separated and transferred to membranes, the resulting membranes were incubated overnight at 4°C with rabbit anti–mouse MCT1 Ab (catalog 20139-1-AP; Proteintech), rabbit anti–mouse BSG Ab (catalog Ab212057; Abcam), rabbit anti–mouse apoptosis-inducing factor Ab (catalog 5318; Cell Signaling Technology), rabbit anti–mouse translocase of outer mitochondrial membrane 20 Ab (catalog 42406; Cell Signaling Technology), rabbit anti–mouse COX IV Ab (catalog 4850; Cell Signaling Technology), rabbit anti–mouse MEK1/2 Ab (catalog 8727; Cell Signaling Technology), rabbit anti–mouse Akt Ab (catalog 4691; Cell Signaling Technology), rabbit anti–mouse phospho-Akt Ab (catalog 4060; Cell Signaling Technology), or rabbit anti–mouse β-actin Ab (catalog 4970; Cell Signaling Technology). Membranes were washed and incubated with HRP-conjugated anti–rabbit IgG Ab (catalog 7074; Cell Signaling Technology). An enhanced chemiluminescence detection system (catalog RPN2106; GE Healthcare) or Immobilon Forte Western HRP Substrate (catalog WBLUF0100; MilliporeSigma) was used to visualize Ab-bound proteins.

### Immunoelectron microscopy.

Preparation for immunoelectron microscopy was performed as described previously (47). In brief, segments of liver and kidney were stained with rabbit anti–mouse BSG Ab (catalog ab212057; Abcam) or with PBS, followed by secondary hybridization with HRP-conjugated goat anti–rabbit IgG F(ab′)_2_ (catalog 424141; Histofine; Nichirei Corporation). The stained tissues then were fixed with 1% glutaraldehyde, incubated with DAB for 30 minutes at room temperature, and washed twice with PBS. Tissues were postfixed in osmium tetroxide, dehydrated in alcohol, and embedded in epoxy resin (Quetol-812; Nissin EM Corporation). Ultrathin sections were examined using a JEM-1400 electron microscope (JOEL, Ltd.).

### Hepatocyte plasma membrane and mitochondrial isolation.

After plating hepatocytes extracted from *Bsg^+/+^* or *Bsg*^–/–^ mice, plasma membrane and mitochondrial isolation were performed using a commercial plasma membrane isolation kit (catalog 9038; Cell Signaling Technology) and a commercial mitochondrial isolation kit for cultured cells (catalog 89874; Thermo Fisher Scientific) according to the manufacturers’ protocols. These samples then were subjected to sodium dodecyl sulfate–polyacrylamide gel electrophoresis and Western blotting.

### Tolerance and loading tests.

For the lactate or pyruvate tolerance tests, fed mice were injected IP with lactic acid solution (0.5 g/kg body weight) (MilliporeSigma) or sodium pyruvate (1.0 g/kg body weight) (MilliporeSigma) formulated in PBS. For the gluconeogenesis assay, the mice were fasted for 16 hours before being administered IP with sodium lactate (2.5 g/kg body weight), sodium pyruvate (2.5 g/kg body weight), alanine (2.5 g/kg body weight) (MilliporeSigma), or l-glutamine (2.5 g/kg body weight) (MilliporeSigma) formulated in PBS. For glucose tolerance tests, *Bsg*^+/+^ and *Bsg*^–/–^ mice fasted overnight were administered glucose (1 g/kg body weight), either by IP injection or by oral gavage. Blood glucose and lactate levels were measured using a glucometer (Johnson & Johnson K.K.) and a lactate test meter (ARKRAY), respectively. Serum pyruvate values were determined using a pyruvate colorimetric assay kit (catalog K609; BioVision, Inc.) according to the manufacturer’s protocol. The *x* axis was used as the baseline of the AUCs in our calculations.

### RNA interference.

For in vitro inhibition of MCT1 (siRNA ID, s73853) or BSG (siRNA ID, s63099), RNA interference was performed using gene expression silencing RNA (Thermo Fisher Scientific). Transfection was performed using Lipofectamine RNAiMAX (Life Technologies, Thermo Fisher Scientific) for siRNA according to the manufacturer’s protocol as described previously (48).

### Lactate production assay.

After plating, C2C12 cells (American Type Culture Collection) transfected with negative control silencing RNA or MCT1 siRNA for 24 hours were exposed to 25 mM d-glucose for the indicated time points. In another experiment, these cells were incubated with 25 mM d-glucose in the absence or presence of 100 nM AZD3965 (Cayman Chemical) for the indicated time points. Spent medium was measured for quantification of lactate production. The lactate production assay was performed using a lactate test meter (ARKRAY).

### Nonbiased comprehensive metabolome analysis.

Intermediates related to the TCA and urea cycles, amino acid metabolism, fatty acid β-oxidation, and ketogenesis in the liver, skeletal muscles, sera, and kidneys of *Bsg^+/+^* or *Bsg^–/–^* male mice were measured by nonbiased comprehensive metabolome analysis. Metabolite extraction from liver, kidney, and serum samples was performed as described previously (49). The resulting solutions were transferred to 5 kDa cutoff centrifugal filter tubes and then used for capillary electrophoresis–time-of-flight mass spectrometry (CE-TOFMS) (Agilent Technologies) analysis. CE-TOFMS–based metabolomic profiling and data analysis were performed as described previously (50, 51). Levels of lactate, pyruvate, and α-ketoglutarate were analyzed by capillary ion chromatography–mass spectrometry as described previously (52).

### ^13^C_3_-labeled pyruvate/lactate–exposed hepatocyte study.

Isolated hepatocytes obtained from adult *Bsg^+/+^* and *Bsg^–/–^* mice were washed twice with 5% mannitol and then incubated for 4 hours in glucose-free Dulbecco’s modified Eagle medium (catalog A14430-01; Thermo Fisher Scientific) supplemented with 20 mM sodium lactate/2 mM pyruvate. These supplements consisted of 40% ^13^C_3_-labeled sodium lactate (catalog CLM-1579-0.5; Cambridge Isotope Laboratories, Inc.)/pyruvate (catalog CLM-2440-0.5; Cambridge Isotope Laboratories, Inc.) and 60% unlabeled sodium lactate/pyruvate (MilliporeSigma). Cell lysates and media were analyzed by CE-TOFMS to determine ^13^C_3_-labeled carbon incorporation into metabolites as previously described (52, 53).

### Hepatocyte glucose production assay.

After plating, hepatocytes were incubated in glucose production buffer consisting of glucose-free Dulbecco’s modified Eagle medium supplemented with 20 mM sodium lactate and 2 mM sodium pyruvate in the absence or presence of 100 nM AZD3965 (Cayman Chemical), 250 μM β-chloro-l-alanine hydrochloride (Tokyo Chemical Industry), 10 mM l-glutamine, and 20 μM EGCG (MilliporeSigma) for 4 hours. Spent medium was collected for quantification of glucose production. The glucose production assay was performed using the commercial Glucose (GO) Assay Kit (catalog GAGO20; MilliporeSigma) according to the manufacturer’s protocol.

### Real-time PCR.

Total RNA was isolated from snap-frozen livers or kidneys using the RNeasy Micro Kit (QIAGEN). Real-time PCR was conducted using the Applied Biosystems Prism 7500HT sequence detection system and TaqMan gene expression assays (Applied Biosystems) (46). The TaqMan probe and primer catalog numbers for the individual genes were as follows: *Bsg*, Mm01144228_g1; *slc16a1* (*Mct1*), Mm01306379_m1; *Pcx*, Mm00500992_m1; *Pck1*, Mm01247058_m1; *G6pc*, Mm00839363_m1; *Cps1*, Mm01256489_m1; *Ass1* Mm00711256_m1; *Echs1* Mm01276347_m1; *CPT1a*, Mm01231183_m1; and *Nrf2*, Mm00477784_m1. Data for these genes were normalized against those for the β-actin–encoding *Actb* gene (catalog Mm02619580_g1). Applied Biosystems Sequence Detection software (version 1.3.1) was used for analysis.

### Measurement of serum levels of ammonia and ALT.

Serum values of ammonia or ALT were measured using commercial assay kits (Abcam and Sanritsu Zelkova Corporation, respectively) according to the respective manufacturers’ protocols.

### Hepatocyte ketone body production assay.

After plating, hepatocytes were incubated for 12 hours in ketone body production buffer consisting of 2.5 mM d-glucose and 400 mM sodium octanoate (MilliporeSigma) in the absence or presence of 4 mM l-glutamine (MilliporeSigma). Spent medium was collected for quantification of 3-hydroxybutyrate production. The 3-hydroxybutyrate production assay was performed using the commercial EnzyChrom Ketone Body Assay Kit (catalog EKBD-100; Bioassay Systems) according to the manufacturer’s protocol.

### Measurement of serum insulin levels.

Serum insulin values were analyzed using a commercial ELISA kit (catalog M1102; Morinaga Institute of Biological Science, Inc.) according to the manufacturer’s protocol.

### Insulin tolerance test.

After 6 hours of food deprivation, animals (*Bsg^+/+^* or *Bsg^–/–^* mice that had been maintained for 16 weeks on HFD) were administered IP with insulin (0.6 units/kg body weight) (MilliporeSigma) (54). Blood glucose levels were measured at 0, 30, 60, 90, and 120 minutes after insulin treatment.

### Hyperinsulinemic-euglycemic clamp technique.

An infusion catheter was inserted into the right jugular veins of 8- to 12-week-old *Bsg*^+/+^ or *Bsg*^–/–^ mice as described previously (55). In brief, only mice that had lost less than 10% of their preoperative weight were examined at 3–5 days after surgery. Each mouse was fasted for 5 hours and allowed to move freely throughout the test. Blood samples were collected, and 35% glucose (Otsuka Pharmaceutical Co. Ltd.) and regular human insulin (Novolin R, 7.5 mU/kg/min; Novo Nordisk Pharmaceutical Industries Inc.) were then infused intravenously. Blood glucose values (Glucometer Elite; Bayer Corp.) were measured at 10-minute intervals. During continuous infusion of insulin, these levels were maintained between 120 and 150 mg/dL by the administration of glucose.

### Measurement of hepatic TGs.

For hepatic TG determination, livers were solubilized by homogenization in a solution of 5% Nonidet P-40 (NP-40) in water; the resulting homogenate was incubated at 80°C in a water bath for 5 minutes until the NP-40 became cloudy, then cooled to room temperature. Samples were centrifuged at 12,000*g* for 2 minutes to remove any insoluble material. TG levels then were measured using the commercial LabAssay Triglyceride kit (catalog 632-50991; FUJIFILM Wako Pure Chemical Co.), as described previously (56).

### Twenty-four-hour respiratory quotient measurements.

Indirect calorimetry was performed using Oxymax chambers (Columbus Instruments). Light period data were collected for 12 hours starting from 0900 hours, and dark period data were collected for 12 hours starting from 2100 hours. All mice were acclimatized for 48 hours before measurements and then evaluated for 44 hours.

### Statistics.

Data are presented as means ± SEM. Statistical analyses were performed using a 2-tailed unpaired Student’s *t* test for single comparisons or using a 1-way ANOVA with post hoc Tukey’s test for multiple comparisons. *P* values less than 0.05 were considered statistically significant. Comparisons were performed using Prism (version 7; GraphPad Software).

### Study approval.

All animal studies were approved by the Animal Experimentation Committee of the Nagoya University Graduate School of Medicine and were conducted according to the Nagoya University Regulations for Animal Experiments.

## Author contributions

AR, T Kosugi, RB, and K Kadomatsu conceived the study; AR, T Kosugi, KM, RB, YG, TI, YS, AH, ST, TO, K Kondoh, KN, KT, TS, NK, TN, HA, T Koike, YM, and SM developed methodology; AR, T Kosugi, RB, YG, KZ, and AH investigated; TI, TH, and YY provided resources; T Kosugi and K Kadomatsu wrote the original draft; T Kosugi, SM, and K Kadomatsu acquired funding; and TS, TI, TN, YY, YM, SM, and K Kadomatsu supervised.

## Supplementary Material

Supplemental data

## Version 1 10/22/2021

Electronic publication

## References

[1] Owen OE (2002). The key role of anaplerosis and cataplerosis for citric acid cycle function. J Biol Chem.

[2] Burgess SC (2006). Diminished hepatic gluconeogenesis via defects in tricarboxylic acid cycle flux in peroxisome proliferator-activated receptor gamma coactivator-1alpha (PGC-1alpha)-deficient mice. J Biol Chem.

[3] Garber AJ (1974). Hepatic ketogenesis and gluconeogenesis in humans. J Clin Invest.

[4] Sunny NE (2011). Excessive hepatic mitochondrial TCA cycle and gluconeogenesis in humans with nonalcoholic fatty liver disease. Cell Metab.

[5] Go Y (2016). Inhibition of pyruvate dehydrogenase kinase 2 protects against hepatic steatosis through modulation of tricarboxylic acid cycle anaplerosis and ketogenesis. Diabetes.

[6] Cappel DA (2019). Pyruvate-carboxylase-mediated anaplerosis promotes antioxidant capacity by sustaining TCA cycle and redox metabolism in liver. Cell Metab.

[7] Basu R (2013). Pathogenesis of prediabetes: role of the liver in isolated fasting hyperglycemia and combined fasting and postprandial hyperglycemia. J Clin Endocrinol Metab.

[8] Yang Q (2018). Metabolites as regulators of insulin sensitivity and metabolism. Nat Rev Mol Cell Biol.

[9] Hughey CC, Crawford PA (2019). Pyruvate carboxylase wields a double-edged metabolic sword. Cell Metab.

[10] Crawford SO (2010). Association of blood lactate with type 2 diabetes: the Atherosclerosis Risk in Communities Carotid MRI Study. Int J Epidemiol.

[11] Juraschek SP (2013). Plasma lactate and diabetes risk in 8045 participants of the atherosclerosis risk in communities study. Ann Epidemiol.

[12] Juraschek SP (2013). Lactate and risk of incident diabetes in a case-cohort of the atherosclerosis risk in communities (ARIC) study. PLoS One.

[13] Halestrap AP, Price NT (1999). The proton-linked monocarboxylate transporter (MCT) family: structure, function and regulation. Biochem J.

[14] Halestrap AP, Wilson MC (2012). The monocarboxylate transporter family--role and regulation. IUBMB Life.

[15] Philp NJ (1998). Monocarboxylate transporter MCT1 is located in the apical membrane and MCT3 in the basal membrane of rat RPE. Am J Physiol.

[16] Kosugi T (2015). CD147 (EMMPRIN/basigin) in kidney diseases: from an inflammation and immune system viewpoint. Nephrol Dial Transplant.

[17] Muramatsu T (2016). Basigin (CD147), a multifunctional transmembrane glycoprotein with various binding partners. J Biochem.

[18] Wilson MC (2005). Basigin (CD147) is the target for organomercurial inhibition of monocarboxylate transporter isoforms 1 and 4: the ancillary protein for the insensitive MCT2 is EMBIGIN (gp70). J Biol Chem.

[19] Kirk P (2000). CD147 is tightly associated with lactate transporters MCT1 and MCT4 and facilitates their cell surface expression. EMBO J.

[20] Yurchenko V (2010). Cyclophilin-CD147 interactions: a new target for anti-inflammatory therapeutics. Clin Exp Immunol.

[21] Tang Y (2005). Extracellular matrix metalloproteinase inducer stimulates tumor angiogenesis by elevating vascular endothelial cell growth factor and matrix metalloproteinases. Cancer Res.

[22] McCommis KS (2015). Loss of mitochondrial pyruvate carrier 2 in the liver leads to defects in gluconeogenesis and compensation via pyruvate-alanine cycling. Cell Metab.

[23] Tompkins SC (2019). Disrupting mitochondrial pyruvate uptake directs glutamine into the TCA cycle away from glutathione synthesis and impairs hepatocellular tumorigenesis. Cell Rep.

[24] Gray LR (2015). Hepatic mitochondrial pyruvate carrier 1 is required for efficient regulation of gluconeogenesis and whole-body glucose homeostasis. Cell Metab.

[25] White HM (2015). The role of TCA cycle anaplerosis in ketosis and fatty liver in periparturient dairy cows. Animals (Basel).

[26] Satapati S (2015). Mitochondrial metabolism mediates oxidative stress and inflammation in fatty liver. J Clin Invest.

[27] Takagi A (2016). Mammalian autophagy is essential for hepatic and renal ketogenesis during starvation. Sci Rep.

[28] Tajima T (2019). β-hydroxybutyrate attenuates renal ischemia-reperfusion injury through its anti-pyroptotic effects. Kidney Int.

[29] Bricker DK (2012). A mitochondrial pyruvate carrier required for pyruvate uptake in yeast, Drosophila, and humans. Science.

[30] Herzig S (2012). Identification and functional expression of the mitochondrial pyruvate carrier. Science.

[31] Brooks GA (2018). The science and translation of lactate shuttle theory. Cell Metab.

[32] Li X (2020). Enhanced glucose metabolism mediated by CD147 contributes to immunosuppression in hepatocellular carcinoma. Cancer Immunol Immunother.

[33] De Bari L (2004). Partial reconstruction of in vitro gluconeogenesis arising from mitochondrial l-lactate uptake/metabolism and oxaloacetate export via novel L-lactate translocators. Biochem J.

[34] Hashimoto T (2006). Colocalization of MCT1, CD147, and LDH in mitochondrial inner membrane of L6 muscle cells: evidence of a mitochondrial lactate oxidation complex. Am J Physiol Endocrinol Metab.

[35] Marchiq I (2015). Genetic disruption of lactate/H+ symporters (MCTs) and their subunit CD147/BASIGIN sensitizes glycolytic tumor cells to phenformin. Cancer Res.

[36] Lou J (2020). Hepatic CD147 knockout modulates liver steatosis and up-regulates autophagy in high-fat-diet-induced NAFLD mice. Biochem Biophys Res Commun.

[37] Carneiro L (2017). AMPK activation caused by reduced liver lactate metabolism protects against hepatic steatosis in MCT1 haploinsufficient mice. Mol Metab.

[38] Jensen NJ (2020). Effects of ketone bodies on brain metabolism and function in neurodegenerative diseases. Int J Mol Sci.

[39] Ruegsegger GN (2019). Insulin deficiency and intranasal insulin alter brain mitochondrial function: a potential factor for dementia in diabetes. FASEB J.

[40] Kirk E (2009). Dietary fat and carbohydrates differentially alter insulin sensitivity during caloric restriction. Gastroenterology.

[41] Luukkonen PK (2020). Effect of a ketogenic diet on hepatic steatosis and hepatic mitochondrial metabolism in nonalcoholic fatty liver disease. Proc Natl Acad Sci U S A.

[42] Igakura T (1998). A null mutation in basigin, an immunoglobulin superfamily member, indicates its important roles in peri-implantation development and spermatogenesis. Dev Biol.

[43] Chen S (2004). Effects of flanking genes on the phenotypes of mice deficient in basigin/CD147. Biochem Biophys Res Commun.

[44] Ito T (2017). Secreted ectodomain of SIGLEC-9 and MCP-1 synergistically improve acute liver failure in rats by altering macrophage polarity. Sci Rep.

[45] Sakamoto K (2019). Glycan sulfation patterns define autophagy flux at axon tip via PTPRσ-cortactin axis. Nat Chem Biol.

[46] Masuda T (2017). Growth factor midkine promotes nuclear factor of activated T cells-regulated T-cell-activation and Th1 cell differentiation in lupus nephritis. Am J Pathol.

[47] Kato N (2009). The E-selectin ligand basigin/CD147 is responsible for neutrophil recruitment in renal ischemia/reperfusion. J Am Soc Nephrol.

[48] Yoshioka T (2019). CD147/basigin deficiency prevents the development of podocyte injury through FAK signaling. Am J Pathol.

[49] Doke T (2018). Lacking ketohexokinase-A exacerbates renal injury in streptozotocin-induced diabetic mice. Metabolism.

[50] Hirayama A (2009). Quantitative metabolome profiling of colon and stomach cancer microenvironment by capillary electrophoresis time-of-flight mass spectrometry. Cancer Res.

[51] Soga T (2009). Metabolomic profiling of anionic metabolites by capillary electrophoresis mass spectrometry. Anal Chem.

[52] Hirayama A (2020). The use of a double coaxial electrospray ionization sprayer improves the peak resolutions of anionic metabolites in capillary ion chromatography-mass spectrometry. J Chromatogr A.

[53] Krycer JR (2017). Dynamic metabolomics reveals that insulin primes the adipocyte for glucose metabolism. Cell Rep.

[54] Maekawa R (2018). Glucose-dependent insulinotropic polypeptide is required for moderate high-fat diet- but not high-carbohydrate diet-induced weight gain. Am J Physiol Endocrinol Metab.

[55] Kubota T (2011). Impaired insulin signaling in endothelial cells reduces insulin-induced glucose uptake by skeletal muscle. Cell Metab.

[56] Ishimoto T (2013). High-fat and high-sucrose (Western) diet induces steatohepatitis that is dependent on fructokinase. Hepatology.

