# Supplementary material for: Basigin deficiency prevents anaplerosis and ameliorates insulin resistance and hepatosteatosis
Source: JCI Insight. 2021 Oct 22;6(20):e142464. doi: 10.1172/jci.insight.142464 (PMC8564892; doi:10.1172/jci.insight.142464)

Supplemental Figure legends

Supplemental Figure 1. Expression of MCT1 and BSG in the liver and kidneys.

(A) Immunofluorescence staining for BSG, MCT1 and MCT4 in skeletal muscles from *Bsg*$^{+/+}$ and *Bsg*$^{-/-}$ mice. Scale bar, 100 µm. **(B)** BSG expression in mitochondria or cytosol of isolated hepatocytes as determined by western blotting. **(C, D)** Expression of the *Slc16a1* gene, which encodes MCT1, in the liver **(C)** and kidneys **(D)** of *Bsg*$^{+/+}$ or *Bsg*$^{-/-}$ mice (n=6-8/genotype). mRNA levels were normalized to those encoding the housekeeping protein β-actin. White columns and circles, *Bsg*$^{+/+}$ mice; gray columns and black circles, *Bsg*$^{-/-}$ mice. Data are presented as means ± SEM. Scatter plots display the data for individual mice. N.S., not significant (p ≥0.05), for the comparison of *Bsg*$^{+/+}$ and *Bsg*$^{-/-}$ at the indicated time point (two-tailed unpaired Student's *t* test). **(E)** Time course of extracellular lactate levels in C2C12 cells transfected with control or MCT1 siRNA and cultured in medium supplemented with 25 mM D-glucose. n=3 for independent experiments. White circles, control siRNA; black circles, MCT1 siRNA. For all relevant panels, data are presented as means ± SEM. **p<0.01, ***p<0.005, for the comparison of siControl and siMCT1 at the indicated time point. **(F)** Time course of extracellular lactate values in the absence or presence of 100 nM AZD3965 (an inhibitor of MCT1 activity). White circles, DMSO; black circles, AZD3965.

Supplemental Figure 2. *Bsg* deficiency leads to the depletion of TCA cycle intermediates in the kidneys.

(A) Static metabolome analysis was performed for TCA cycle intermediates in renal cortex in *Bsg*$^{+/+}$ or *Bsg*$^{-/-}$ animals subjected to fasting conditions (n=7-8/genotype). White columns and circles, *Bsg*$^{+/+}$ mice; gray columns and black circles, *Bsg*$^{-/-}$ mice. Data are presented as means ± SEM. Scatter plots display the data for individual mice. N.S., not significant (p ≥0.05), for

the comparison of *Bsg*$^{+/+}$ and *Bsg*$^{-/-}$ at the indicated time point (two-tailed unpaired Student's *t* test).

Supplemental Figure 3. *Bsg* deficiency impairs gluconeogenesis in vivo.

(A) Blood glucose levels of *Bsg*$^{+/+}$ and *Bsg*$^{-/-}$ mice under feeding conditions (n=12/genotype). White columns and circles, *Bsg*$^{+/+}$ mice; gray columns and black circles, *Bsg*$^{-/-}$ mice. Data are presented as means ± SEM. Scatter plots display the data for individual mice. **(B, C)** Expression of transcripts encoding proteins involved in gluconeogenesis, including pyruvate carboxylase (PCX), phosphoenolpyruvate carboxykinase (PCK)-1, and glucose-6-phosphatase (G6PC), in the liver **(B)** and kidneys **(C)** of *Bsg*$^{+/+}$ or *Bsg*$^{-/-}$ mice under feeding and fasting conditions (n=6/condition). mRNA levels were normalized to those encoding the housekeeping protein β-actin.

Supplemental Figure 4. Static metabolome analysis in vivo.

Static metabolome analysis for amino acids in renal cortex **(A)**, sera **(B)**, and ureagenesis-related metabolites in the liver **(C)** from *Bsg*$^{+/+}$ or *Bsg*$^{-/-}$ mice under fasting conditions (n=7-8/genotype and condition combination). White columns and circles, *Bsg*$^{+/+}$ mice; gray columns and black circles, *Bsg*$^{-/-}$ mice. Data are presented as means ± SEM. Scatter plots display the data for individual mice.

Supplemental Figure 5. Improvement of diet-induced insulin resistance in *Bsg*-deficient mice.

(A) Body weights of *Bsg*$^{+/+}$ and *Bsg*$^{-/-}$ mice maintained on CD or HFD for 16 weeks. White circles, HFD-fed *Bsg*$^{+/+}$ mice; black circles, HFD-fed *Bsg*$^{-/-}$ mice; white squares, CD-fed *Bsg*$^{+/+}$ mice; black squares, CD-fed *Bsg*$^{-/-}$ mice (n=10-11/genotype and diet combination). **(B)** Total

energy intake of *Bsg*$^{+/+}$ and *Bsg*$^{-/-}$ animals over the 16-week study period (n=10-11/genotype and diet combination). White columns, *Bsg*$^{+/+}$ mice; gray columns, *Bsg*$^{-/-}$ mice. **(C, D)** Blood glucose excursions and the AUC scores in *Bsg*$^{+/+}$ and *Bsg*$^{-/-}$ mice (maintained on CD or HFD for 16 weeks) during **(C)** lactate tolerance tests (n=6-12) and **(D)** pyruvate tolerance tests (n=8-12). **(E)** Serum triglycerides levels of *Bsg*$^{+/+}$ and *Bsg*$^{-/-}$ mice at 16 weeks. n=5-6/genotype and diet combination. **(F)** 3-hydroxybutyrate contents in the liver and kidneys of *Bsg*$^{+/+}$ and *Bsg*$^{-/-}$ mice maintained on CD or HFD for 16 weeks (n=5-6). **(G)** Twenty-four-hour respiratory quotient (PER; VO_2/VCO_2) and volume of oxygen consumption (VO_2) as measured by indirect calorimetry with *ad libitum* access to HFD and water. n=6. **(H)** Liver levels of the transcript encoding NF-E2-related factor (NRF)-2 in HFD-fed *Bsg*$^{+/+}$ and *Bsg*$^{-/-}$ mice (n=5-6/genotype). For all panels, data are presented as means ± SEM. *p<0.05, **p<0.01, ****p<0.0001, N.S., not significant (p ≥0.05), for the comparison of *Bsg*$^{+/+}$ and *Bsg*$^{-/-}$ at the indicated time point (two-tailed unpaired Student's *t* test).

Supplemental Figure 1

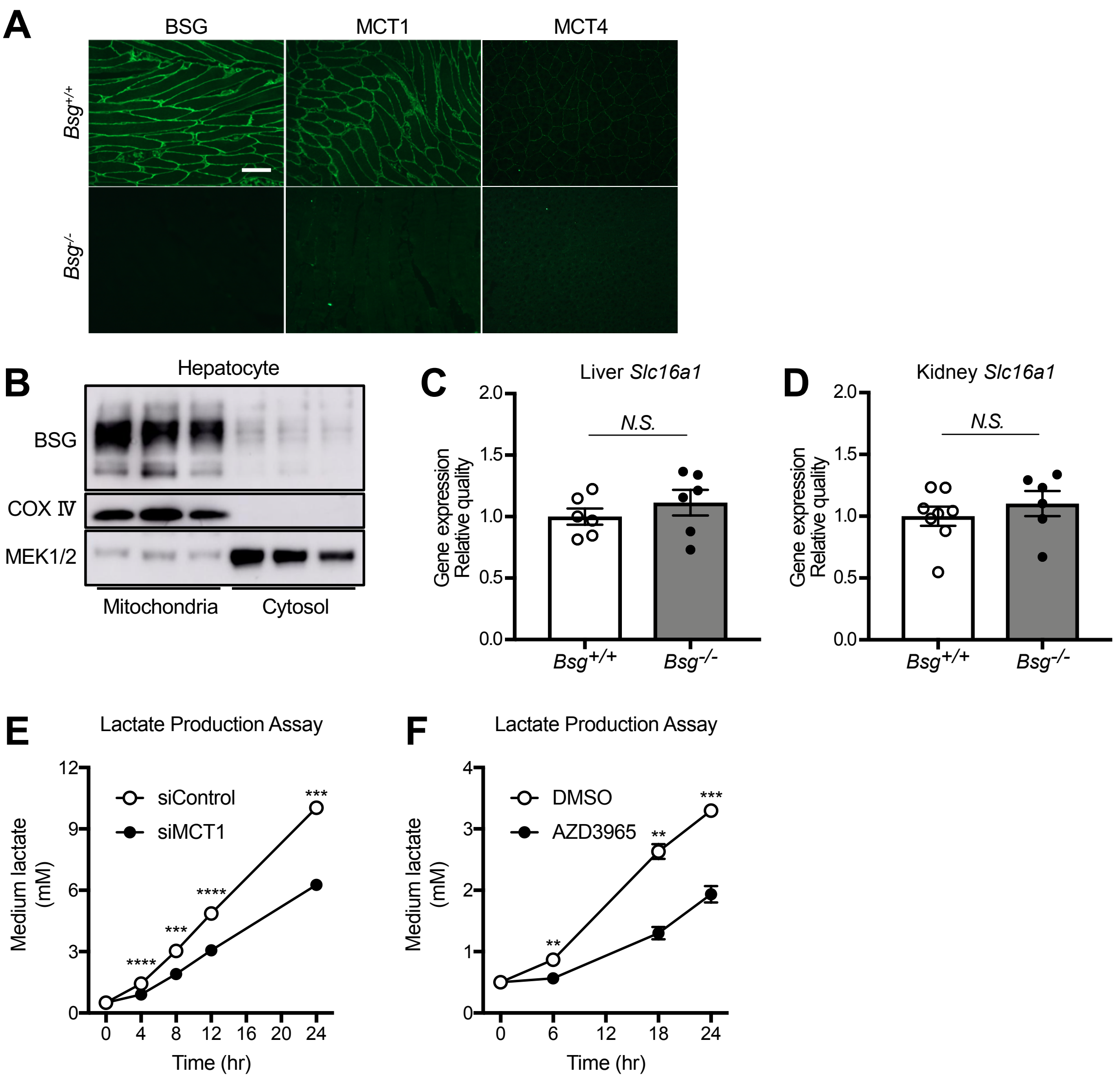

Supplemental Figure 2

A

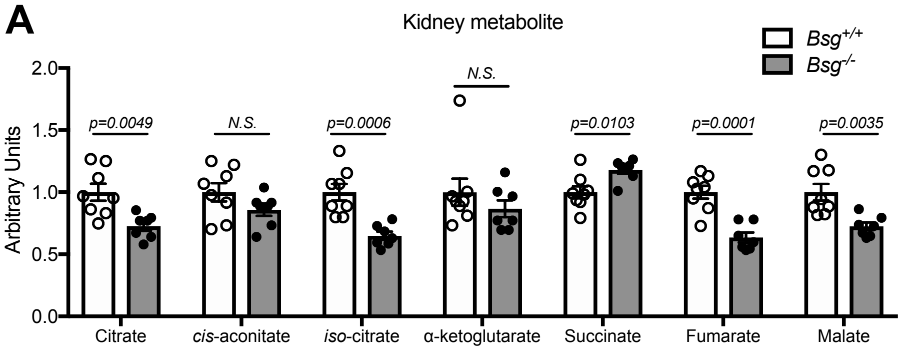

Supplemental Figure 3

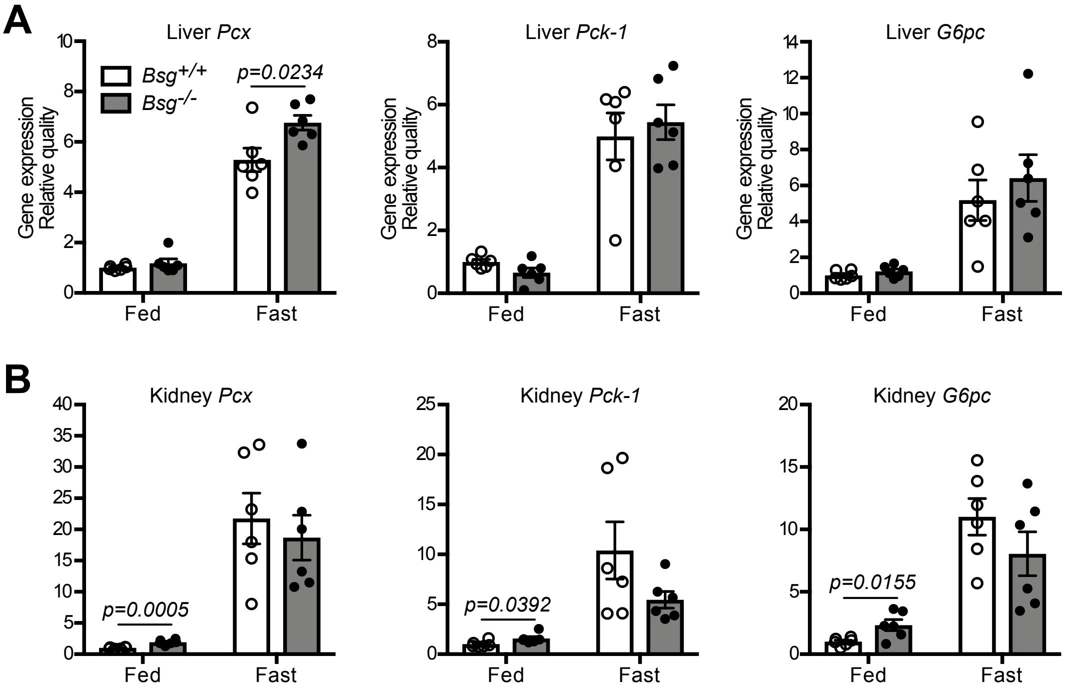

Supplemental Figure 4

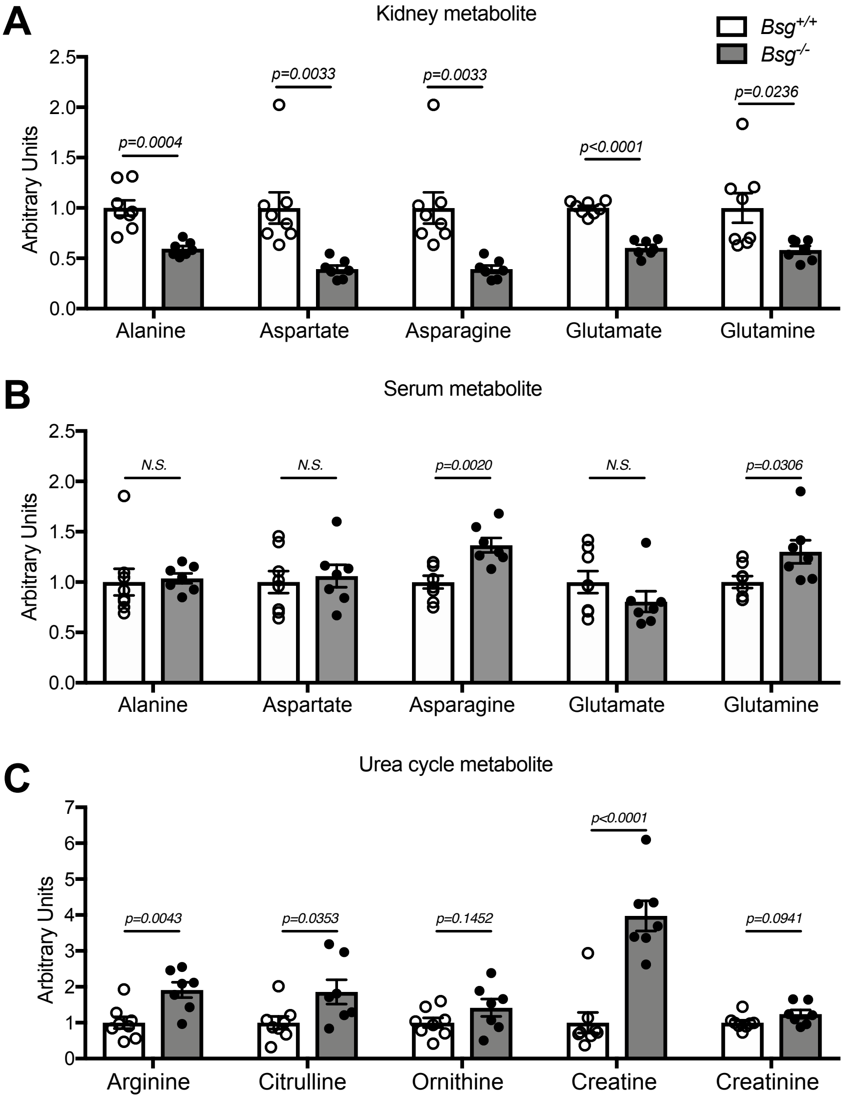

Supplemental Figure 5

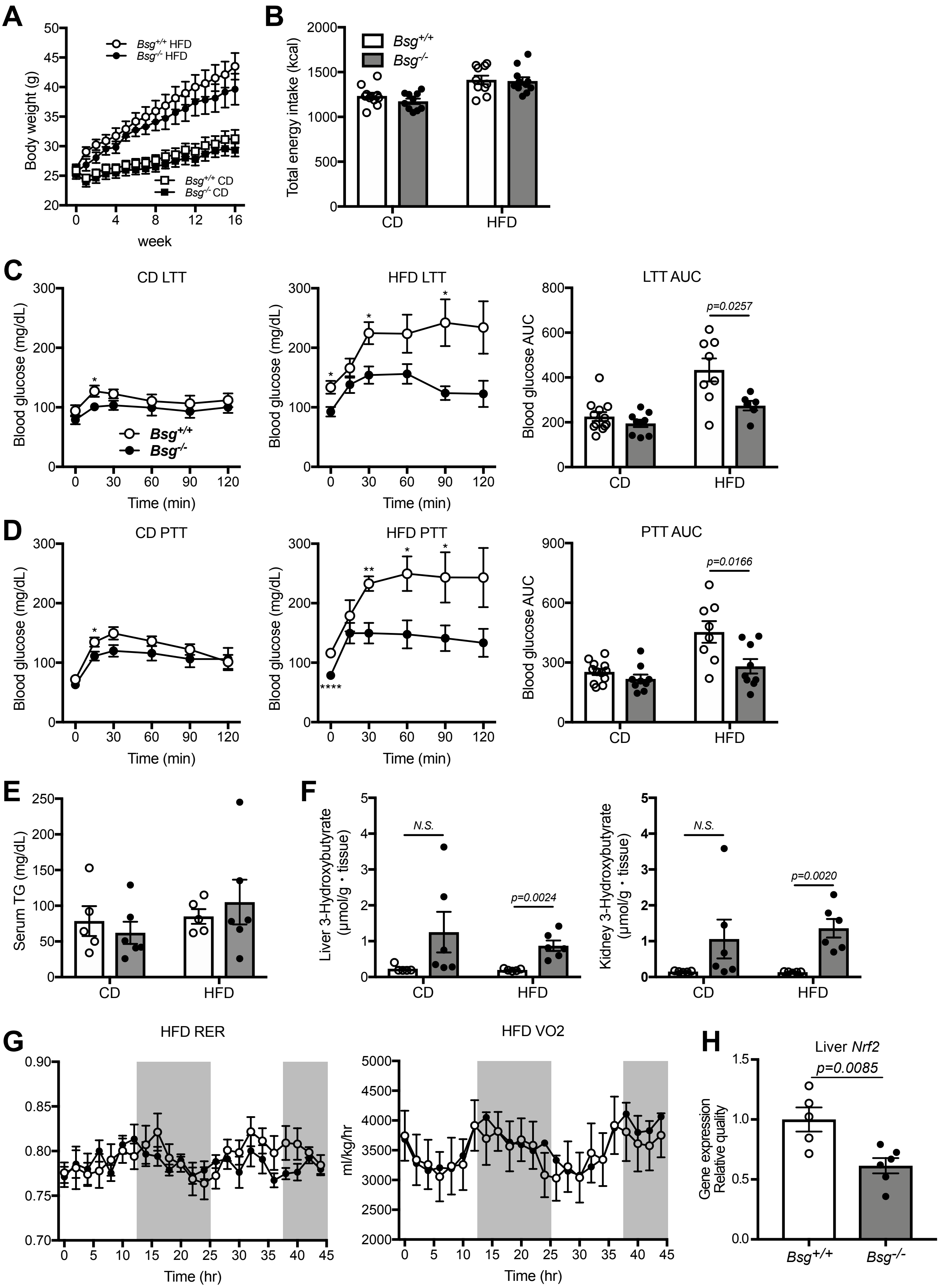

Supplement: Supplemental data [file jciinsight-6-142464-s056.pdf]
